# A Recombinant Fungal Lectin for Labeling Truncated Glycans on Human Cancer Cells

**DOI:** 10.1371/journal.pone.0128190

**Published:** 2015-06-04

**Authors:** Aymeric Audfray, Mona Beldjoudi, Adrien Breiman, Amandine Hurbin, Irene Boos, Carlo Unverzagt, Mourad Bouras, Sylvie Lantuejoul, Jean-Luc Coll, Annabelle Varrot, Jacques Le Pendu, Benoit Busser, Anne Imberty

**Affiliations:** 1 CERMAV, UPR5301, CNRS, University Grenoble Alpes, 38041 Grenoble, France; 2 IAB, University Grenoble Alpes, F-38000 Grenoble, France; 3 INSERM U823, IAB, F-38000 Grenoble, France; 4 University El Hadj Lakhdar, 05000 Batna, Algeria; 5 INSERM, UMR892, 44007 Nantes, France; 6 CNRS, UMR6299, 44007 Nantes, France; 7 IRS UN, University of Nantes, Nantes, France; 8 Nantes University Hospital, 44000 Nantes, France; 9 Bioorganische Chemie, Gebäude NW1, Universität Bayreuth, 95440 Bayreuth, Germany; 10 Grenoble University Hospital, F-38000 Grenoble, France; Ghent University, BELGIUM

## Abstract

Cell surface glycoconjugates present alterations of their structures in chronic diseases and distinct oligosaccharide epitopes have been associated with cancer. Among them, truncated glycans present terminal non-reducing β-N-acetylglucosamine (GlcNAc) residues that are rare on healthy tissues. Lectins from unconventional sources such as fungi or algi provide novel markers that bind specifically to such epitopes, but their availability may be challenging. A GlcNAc-binding lectin from the fruiting body of the fungus *Psathyrella velutina* (PVL) has been produced in good yield in bacterial culture. A strong specificity for terminal GlcNAc residues was evidenced by glycan array. Affinity values obtained by microcalorimetry and surface plasmon resonance demonstrated a micromolar affinity for GlcNAcβ1-3Gal epitopes and for biantennary N-glycans with GlcNAcβ1-2Man capped branches. Crystal structure of PVL complexed with GlcNAcβ1-3Gal established the structural basis of the specificity. Labeling of several types of cancer cells and use of inhibitors of glycan metabolism indicated that rPVL binds to terminal GlcNAc but also to sialic acid (Neu5Ac). Analysis of glycosyltransferase expression confirmed the higher amount of GlcNAc present on cancer cells. rPVL binding is specific to cancer tissue and weak or no labeling is observed for healthy ones, except for stomach glands that present unique αGlcNAc-presenting mucins. In lung, breast and colon carcinomas, a clear delineation could be observed between cancer regions and surrounding healthy tissues. PVL is therefore a useful tool for labeling agalacto-glycans in cancer or other diseases.

## Introduction

Changes in cell surface glycosylation are known to be associated with a large number of chronic diseases and the cancer glycans represent a very promising field for biomarker discovery [1–3]. Correlation of inflammation or metastasis with overexpression of sialylated and fucosylated epitopes, such as sialyl Lewis x has been the subject of intensive research. However, in other cases, the very active metabolism and divison of cancer cells can result in the abnormal exposure of cryptic epitopes. This internal part of glycoconjugates, that would have been decorated by other monosaccharides in normal cells, may therefore be exposed on the cell surface and become available for detection by antibodies or lectins [4].

**Fig 1:**
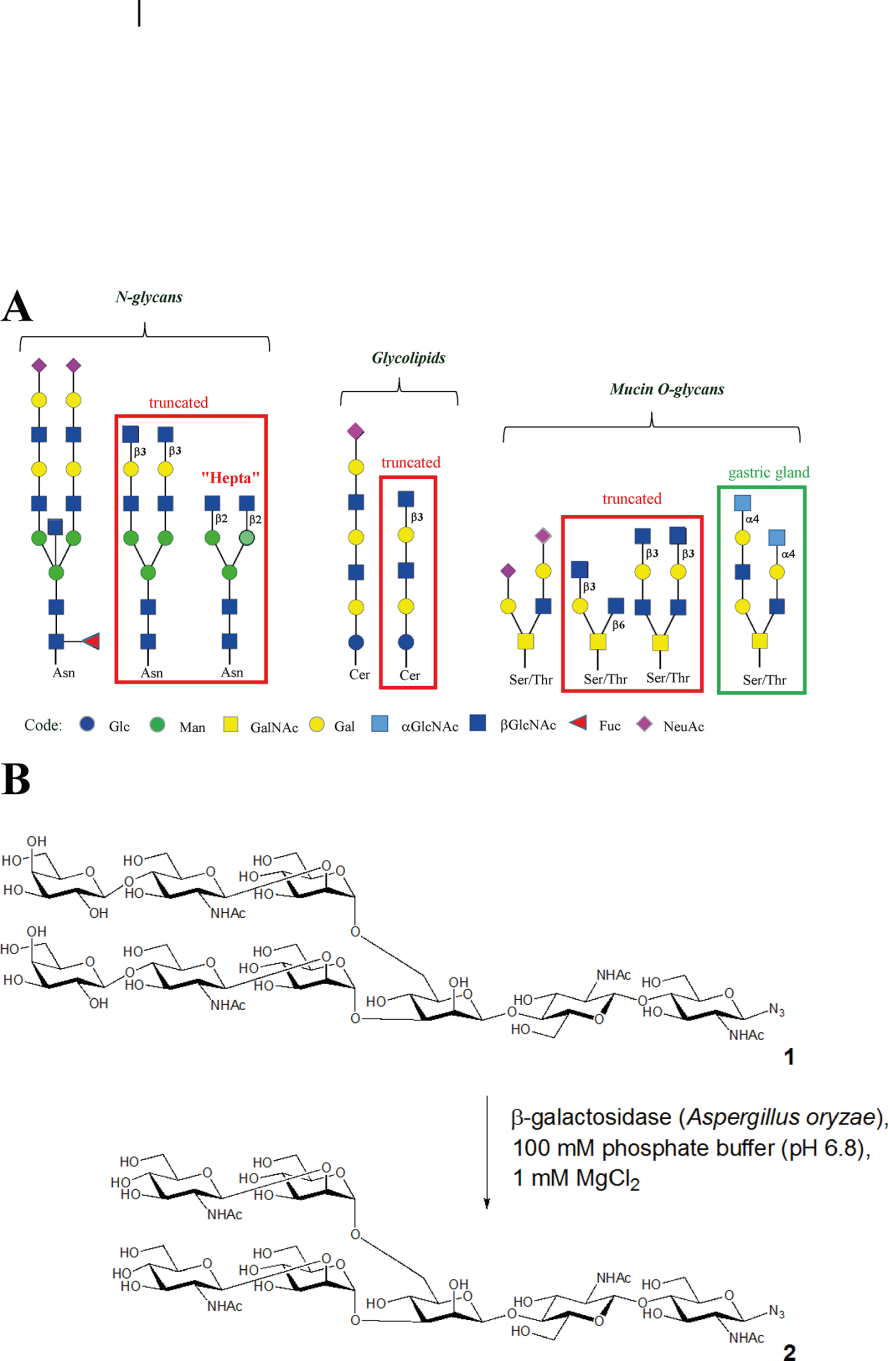
Representation of glycoconjugates and binding of rPVL to glycans on chips. A. Examples of normal and truncated oligosaccharides that can be found on normal or cancer tissues. Coding for schematic representation of monosaccharides is in the lower part of the figure. The heptasaccharide used in binding experiments is indicated as “hepta”. B. Synthesis of heptasaccharide azide 2 corresponding to oligosaccharide “hepta” in panel A.

N-acetylglucosamine (GlcNAc) is a carbohydrate residue that is present in the inner part of N-glycans, as the core chitobiose linked to asparagine residue and more rarely as β1–4 linked to the core branched mannose in bisected N-glycans. GlcNAc is also present in the branches of these glycoconjugates, either β1-2-linked to mannose (Man) on the trimannose core or β1-3-linked to galactose (Gal) as part of polylactosamine extended branches (Fig 1A). Nevertheless, due to the activity of galactosyltransferases and sialyltransferases, these GlcNAc residues are not in terminal positions in normal tissues. Similarly glycosphingolipids contain GlcNAcβ1–3 or β1–6 linked to Gal but not in exposed terminal positions. The epitopes are similar in mucins with Gal, sialic acid (Neu5Ac) or fucose (Fuc) residues capping the terminal epitopes. The only exception is a rare epitope consisting of α1–4 linked GlcNAc-terminated mucins from glandular mucous cells in the stomach [5] (Fig 1A).

The occurrence of aberrant βGlcNAc-terminating epitopes has been observed in a limited number of cancers, including human leukemia cells [6]. IgGs with truncated N-glycans have been reported in the serum of prostate cancer patients [7]. Altered mucins with terminal GlcNAc motifs were proposed to form the Tk epitope, a colon tumor associated antigen [8]. The most precise characterization of the alterations of glycosylation with exposure of internal GlcNAc was performed by glycome analysis of different carcinoma, pointing to the occurrence of short truncated N-glycans terminated by GlcNAβ1-2Man on both antenna and of GlcNAc-terminated linear and branched glycosphingolipids, particularly in lung small-cell carcinoma and adenocarcinoma, but also in kidney, breast and ovary carcinoma [9] (Fig 1A).

Lectins, generally derived from plants, have been demonstrated to be very efficient for detecting aberrant glycosylation in biological samples. A new technology is the use of lectin arrays that can contain lectins with large spectra of specificity and therefore characterize variation of glycosylation [10]. The use of lectins extracted from natural organisms may be time-consuming and may generate problems of contamination or variations between batches. Therefore, recombinant lectin technology is starting to be developed [11]. Lectins that are classically used for GlcNAc-binding are extracted from plants such as wheat (WGA), tomato (SLT) and *Griffonia simplicifolia* (GSL-II). A new GlcNAc-specific lectin (PVL) was purified from the fruiting bodies and mycelium of a fungus, *Psathyrella velutina* [12] and then structurally characterized [13]. A closely related protein, lectin 2 from mushroom *Agrocybe aegerita* (AAL-2) has been recently cloned and demonstrated to bind to hepatoma cells [14]. The PVL structure presents a novel fold among lectins, with seven β-sheets arranged in a β-propeller fold. The lectin therefore adopts a donut shape with six GlcNAc binding sites, and is expected to present strong avidity for cell surfaces presenting high density of terminal GlcNAc residues.

The aim of the present work was to produce a recombinant form of PVL and to characterize its fine specificity towards the different GlcNAc-terminated epitopes that could be present on pathology-related truncated forms of human glycoconjugates. The avidity of the lectin for GlcNAc-decorated surface was evaluated on arrays. Finally, the ability of the lectin to label cancer cells and carcinoma tissues was also described.

## Materials and Methods

### Material

GlcNAc has been purchased from Sigma-Aldrich. Disaccharide GlcNAcβ1-3Gal, lacto-N-tetraose (Galβ1-3GlcNAcβ1-3Galβ1-4Glc), lacto-N-triose (GlcNAcβ1-3Galβ1-4Glc) chitobiose (GlcNAcβ1-4GlcNAc), chitopentaose (GlcNAcβ1-4GlcNAcβ1-4GlcNAcβ1-4GlcNAcβ1-4GlcNAc) have been purchased from Elicityl (Crolles, France). Sialidase from *Clostridium perfringens* has been purchased from Sigma-Aldrich (ref. N2876) or New England Biolabs (Ipswich, MA). ß-D-N-Acetyl-hexosaminidase_f_ from *Streptomyces plicatus* was purchased from New England Biloabs. The biotin-labeled sialic acid-specific lectin MAH was purchased from Vector Labs (Burlingame, CA)

### Synthesis of N-glycan 2

Nonasaccharide azide **1** (Fig 1B) was prepared from egg yolk followed by enzymatic hydrolysis and azidation [15, 16]. 5.1 mg (3.1 μmol) of nonasaccharide **1** were disolved in 203 μl of phosphate buffer (100 mm, pH 6.8, 1 mm MgCl_2_). 10 units of lyophilized β-galactosidase (EC 3.2.1.23) were added to the solution. The mixture was incubated at 37°C for 3 days (TLC: 2-propanol, 1 m ammonium acetate, 2:1). After lyophilization, the residue was purified by gel filtration (Superdex 30, 1.6 x 60 cm, 0.1 m NH_4_HCO_3_, 0.9 ml min^-1^). The peak, eluting at 86 min, was lyophilized and desalted by gel filtration (Sephadex G-25, 2.5 x 15.5 cm, 5% ethanol in water, 0.6 ml min^-1^). The peak, eluting at 75 min, was lyophilized yielding 2.9 mg (2.2 μmol) of heptasaccharide **2** (70%). [α]_D_^22^ = -9.0 (c = 0.5, H_2_O). The purity was confirmed by 360 MHz ^1^H NMR in D_2_O (S1 Fig) ^1^H-NMR (360 MHz, D_2_O): δ = 5.03 (d, *J*_1,2_ < 1 Hz, 1H, H-1^4^), 4.83 (d, *J*_1,2_ < 1 Hz, 1H, H-1^4´^), 4.68–4.64 (m, 2H, H-1^1^, H-1^3^), 4.53 (d, *J*_1,2_ = 7.7 Hz, 1H, H-1^2^), 4.47 (d, *J*_1,2_ = 8.3 Hz, 2H, H-1^5^, H-1^5’^), 4.18–4.15 (m, 1H, H-2^3^), 4.12–4.09 (m, 1H, H-2^4^), 4.04–4.01 (m, 1H, H-2^4’^), 1.99 (s, 3H, NAc), 1.97 (s, 9H, NAc). ESI-MS: m/z calcd for C_50_H_83_N_7_O_35_: 1341.49; found 1342.62 (M+H)^+^, 1365.86 (M+Na)^+^

### Production of recombinant PVL

A nucleotide sequence coding for the peptide sequence of lectin from fruiting body of mushroom *P*. *velutina* (GenBank, accession number DQ232759) [13] supplemented at N-terminal position with the amino-acids MSVVVIS was synthesized after codon optimization for expression in *Escherichia coli* (GenScript, Piscataway, NJ). It was introduced into the expression vector pET25b using NdeI and XhoI restriction sites. The pET25-rPVL vector was transformed into *E*. *coli* BL21(DE3) (Novagen), and cells harboring pET25-rPVL plasmid were grown in LB broth containing 100 μg ml^-1^ ampicillin at 37°C until A600 reached 0.7. The cells were then cultured for 16 h after addition of 0.5 mM isopropyl-β-D-thiogalactopyranoside. After centrifugation (7000 x g for 15 min), bacteria were resuspended in equilibration buffer (20 mM Tris/HCl, pH 7.5, 150 mM NaCl) and broken by cell disruption at a pressure of 1.7 kilobars (Constant systems Ltd). After centrifugation (50000 x g, 30 min at 4°C) and filtration, affinity chromatography on a GlcNAc-agarose column (EY laboratories inc.) was performed on the supernatant. rPVL was allowed to bind to immobilized GlcNAc in equilibration buffer, and after washing (20 mMTris/HCl pH 7.5, 1 M NaCl), it was eluted with 200 mM of free GlcNAc in equilibration buffer. Purified protein was dialyzed extensively against ultrapure water for 7 days, freeze-dried, and stored at 4°C.

### Glycan array

Purified rPVL were labeled with Alexa Fluor 488 (Invitrogen) according to the manufacturer’s instructions and repurified on a D-Salt polyacrylamide desalting column (Pierce). Alexa-labeled rPVL was used for glycan array screening with the standard procedure of the Protein-Glycan Interaction Core (H) of the Consortium for Functional Glycomics.

### Thermal shift analysis

Thermal shift assays were performed using a Mini Opticon, Real Time PCR machine (Bio-Rad Laboratories) with 0.5 mg ml^-1^ of protein diluted in 100 mM NaCl, 20 mM Tris pH 7.5, 100 μM CaCl_2_, with or without oligosaccharide in a total reaction volume of 25 μl. SYPRO Orange (Molecular Probes, CA) was used as a fluorescent probe detected at 530 nm. The temperature was raised using 1°C/minute steps from 25°C to 100°C and fluorescence readings were taken at each interval. A positive ΔTm value indicates that the ligand stabilizes the protein from denaturation, and therefore binds to the protein. A minimum of two independent measurements were performed for each condition

### Microcalorimetry

Recombinant lyophilized rPVL was dissolved in buffer (20 mM Tris/HCl, pH 7.5, NaCl 150 mM, 100 μM CaCl_2_) and degassed. The protein concentration was checked by measuring A280 by using a theoretical molar extinction coefficient of 65,890 M^-1^ cm^-1^. Carbohydrate ligands were dissolved in the same buffer, degassed, and loaded in the injection syringe. ITC was performed with a ITC200 microcalorimeter (GE Healthcare). The rPVL solution was placed in a 200 μl sample cell at 25°C. Titration was performed with 20 injections of 2 μl carbohydrate ligands every 120 s. The experimental data were fitted to a theoretical titration curve using Origin software supplied by GE Healthcare, with ΔH (enthalpy change), Ka (association constant) and n (number of binding sites per monomer) as adjustable parameters. Free energy change (ΔG) and entropy contributions (TΔS) were derived from the equation ΔG = ΔH – TΔS = —RT ln Ka (with T as the absolute temperature and R = 8.314 J mol^-1^ K^-1^). Two independent titrations were performed for each tested ligand.

### Surface plasmon resonance

All SPR experiments were performed on a Biacore X100 instrument (GE Healthcare) at 25°C in HBS (10 mM Hepes/NaOH, pH 7.5, 150 mM NaCl, 0.05% Tween 20) supplemented with 100 μM of CaCl_2_ at a flow rate of 30 μl min^-1^. Binding was measured as resonance units over time after blank subtraction, and data were then evaluated by using the Biacore X100 evaluation software, version 2.0. Dissociation constants were determined by plotting response at equilibrium (Req, 10 s before the end of injection) against analyte concentration. For protein coated chip, 3500 resonance units of rPVL (100 μg ml^-1^, 10 mM acetate buffer pH 6.2) have been immobilized on flow channel 2 of a research grade CM5 chip using standard amine coupling procedures. Flow channel 1 has been activated/deactivated. Experiments consist of injection (association 360 s, dissociation 400 s) of various concentrations of oligosaccharides (2 fold cascade dilutions, from 0 to 10 μM) on both channels.

### Protein crystallography

Crystals of rPVL complexed with GlcNAcβ1-3Gal were obtained by the hanging drop at 20°C. Lyophilized protein was dissolved at 2.5 mg ml^-1^ in 10 mM Hepes/NaOH buffer, pH 7.5, NaCl 150 mM, 100 μM CaCl_2_ and incubated with 1 mM GlcNAcβ1-3Gal during 1h at room temperature prior to co-crystallization. One big rectangular-like crystal was obtained from 20% PEG3350, 0.2 M Sodium Formate and 0.1 M diammonium phosphate after several months. One piece was directly mounted in a cryoloop and flash-freezed in liquid nitrogen. Diffraction data were collected at 100 K at the European Synchrotron Radiation Facility (Grenoble, France) at station BM30A using a ADSC Q315r CCD detector [17]. The data were processed using XDS [18]. All further computing was performed using the CCP4 suite [19] Data quality statistics are summarized in S1 Table. The molecular replacement technique was used to solve the structure with PHASER [20] and the coordinates of the native PVL (Protein Data Bank code 2C4D) [13]. The structure was refined by restrained maximum likelihood refinement using REFMAC 5.8 [21] iterated with manual rebuilding in Coot [22]. Incorporation of the ligand was performed after inspection of the mFo-DFc weighted maps. Water molecules, introduced automatically using Coot, were inspected manually. The stereochemical quality of the models was assessed with the program Molprobity [23], and coordinates were deposited in the Protein Data Bank under codes 4UP4.

### Cells lines

Human non-small cell lung cancer (NSCLC) (H358, A549, H441 and H322), bronchial epithelial (HBEC-3KT), breast cancer (MCF-7, MDA-MB-231), ovarian cancer (OVCAR3), prostate cancer (DU-145), skin squamous cell carcinoma (A431), melanoma (A375, Colo829 and SKMel28), and colon cancer (HT-29) cell lines were obtained from the American Type Culture Collection (ATCC, Manassas, VA). The M119 melanoma cell line was a gift from Dr Nathalie Labarrière (Inserm U892, Nantes, France). The cells were maintained in RPMI-1640 medium (Gibco, Cergy Pontoise, France), except MDA-MB-231 and HT-29, which were grown in DMEM 4.5 g l^-1^ glucose (Gibco), and HBEC-3KT, which were maintained in Keratinocyte-SFM medium (Gibco). All the culture media were supplemented with 10% heat-inactivated fetal bovine serum (5% for MDA-MB-231) and cells were kept in a humidified atmosphere with 5% CO2, at 37°C.

### Flow cytometry

Cells were trypsinized, aliquoted, then washed twice in PBS (with Ca^2+^ and Mg^2+^). After incubation with rPVL-Alexa488 (5 and 10 μg ml^-1^), cells were washed twice and analysis was performed on an Accuri C6 flow cytometer using the CFlow-Plus Software (BD Biosciences). Alternatively, biotinylated rPVL or MAH were used, followed by Streptavidin-phycoerythrin (BD) and analysis was performed on a FACSCalibur with the Cellquest software (BD). Where indicated, cells were pre-teated with sialidase (Sigma) or ß-D-N-acetyl-hexosaminidase for 4h at 37°C.

### Inhibition of glycosylation

A549 cells were seeded in 6 well plates and treated with different glycosylation inhibitors: a fucosyl transferase inhibitor, 2-fluoro-peracetyl fucose (2FF) and a sialyl-transferase inhibitor, 3-fluoro-peracetyl-neuraminic acid (3F-Neu5Ac) that have been described recently [24]. Treatment was performed for 4 days with 400 μM and 100 μM of 2FF and 3F-Neu5Ac respectively. Medium was replaced and inhibitors were added again after 2 days.

In order to investigate the type of glycosylated ligands that are recognized by rPVL, we used Kifunensin (Sigma) which blocks ER α-mannosidase I and consequently the processing of N-glycans; benzyl-2 acetamido-2 deoxy-alpha-D-galactopyranoside (Benzyl-GalNAc; Sigma) which blocks the O-glycosylation process and 1R,2R-(+)-1-phenyl-2-palmitoylamino-3-N-morpholine-1-propanol (PPMP; Avanti Polar Lipids, Alabaster, USA) which inhibits synthesis of glycolipids. Cells were treated with 5 μM Kifunensin for 4 days as above. For Benzyl-GalNAc and PPMP, cells were left in contact with the inhibitors, at 6 mM and 10 μM respectively, over a 24h period, then the medium was changed and the cells were incubated for 1 more day before analysis. As 2FF, 3F-Neu5Ac and PPMP are diluted in DMSO, pure DMSO was added to the medium of control wells in order to have the same DMSO concentration in each well.

### Immunofluorescence analysis

Cells were seeded into 4-chamber culture slides (4x10^4^ cells per chamber). After 24h, cells were washed with ice-cold PBS and fixed with 2% paraformaldehyde for 10 min at 4°C. Before and after incubation with 1% BSA/PBS (w/v), cells were washed with cold PBS three times for 5 min each. Cells were then subjected to immunofluorescence staining with rPVL-Alexa488 for 1h at room temperature and then washed with cold PBS three times for 3 min each. Cells were examined using a BX41 microscope equipped with a DP-70 digital camera system (Olympus, Tokyo, Japan) and a pseudo-confocal microscope ApoTome equipped with AxioCam MRm (N/B).

### Tissue sections

Ethanol-fixed human trachea, esophagus, duodenal junction, jejunum, colon, pancreas, liver, ovary, uterus and vagina samples were obtained from organ donors. 18 tissue samples from 10 different individuals were used to prepare a tissue microarray (TMA) of healthy tissues. Ethanol-fixed colorectal tumor sections were obtained after cancer surgery. Formalin-fixed paraffin-embedded human NSCLC samples (n = 3 squamous cell carcinomas, n = 3 adenocarcinomas) were also obtained. A breast tumor and a lung tumor TMA (formalin-fixed; 40 tumors, 10 paired metastasis and 10 paired adjacent “normal” tissue) were bought from SuperBiochip (Seoul, South Korea). Formalin-fixed canine mammary tumors were obtained from "laboratory of animal histopathology of the Nantes Veterinary School, ONIRIS) Immunostaining analysis was performed on 3-μm-thick tissue sections.

### Histochemistry

The tissue sections were deparaffinized, then endogenous peroxidases were blocked by incubating the sections with PBS containing 3% hydrogen peroxide (v/v), for 5 mn. The sections were then blocked with BSA 5% (w/v) for 30 min at room temperature, followed by incubation with 0,7–1 μg ml^-1^ biotinylated rPVL 1h at room temperature (Ethanol-fixed sections) or 2 μg ml^-1^ of biotinylated rPVL in PBS at 4°C for 18h (for formalin-fixed sections). After washing the sections twice with PBS, an indirect biotin-streptavidin system and DAB (Ventana Medical Systems) or AEC (Vector laboratories) detection kits were used according to the manufacturer’s instructions. The developed slides were washed twice with PBS and counterstained with hematoxylin. After washing with water, the sections were dehydrated and mounted. Sections were observed under a BX41 microscope equipped with a DP-70 digital camera system (Olympus, Tokyo, Japan) or imaged with a NanoZoomer slide-scanner (Hamamatsu, Hamamatsu City).

For treatment with glycosidases, sections were incubated (after deparaffination and hydrogen peroxide blocking) with 50 U of sialidase (New England Biolabs) or 25 U of ß-D-N-acetyl-hexosaminidase for 2 hours at 37°C. Fresh enzymes were then added and the slides were further incubated overnight at 37°C. Control slides were prepared in parallel with the corresponding enzyme buffers (Sodium Citrate pH 6 and 4.5 respectively). After overnight incubation, the slides were washed twice in PBS, blocked with PBS-5% BSA (w/v), stained with rPVL and imaged as described above.

### Ethics statements

All the human ethanol-fixed tissues tissues were collected and stored before the law 88–138 of December 20, 1988 concerning resection of human tissues after death for scientific investigations. For this reason, no approval by a Research Ethics Committee applies. The samples were obtained from the Nantes University Hospital Center for Biological Resources (http://relib.fr), under the Cancerology program approved by the ministry of research (approval DC-2011-1399). Tissue banking and research conduct of the formalin-fixed human tissues were approved by the ministry of research (approval AC-2010-1129) and by the regional Institutional Review Board Comité de Protection de Personnes (CPP) 5—Sud Est (regional board). All patients enrolled in this trial provided written informed consent. These specific samples were previously used as described in literature [25, 26]. Regarding the Tissue MicroArrays obtained from Superbiochips Laboratories Ltd (Seoul, Korea), the company certifies that the “human material have been removed or collected with the donor’s prior consent and that no payment whatsoever has been made to the latter”. Formalin-fixed tissues from canine breast tumors were obtained from "laboratory of animal histopathology” of the Nantes Veterinary School (ONIRIS). Cases coming from the Pathology Laboratory of the Veterinary Teaching Hospital of Oniris were used in this study. The owners’ written consent and approval from the Oniris College of Veterinary Medicine local Animal Welfare Committee were obtained prior to inclusion. The animal care and used protocol adhered to the European Directive number 2010/063 and to the national French regulation (Décret n°2013–118 du 1er février 2013 relatif à la protection des animaux utilisés à des fins scientifiques).

## Results

### Production and characterisation of rPVL

PVL has been produced recombinantly (rPVL) in *E*. *coli* and purified in one step on a GlcNAc-agarose column with a final yield of 1 mg l^-1^ of culture. When assayed on rabbit erythrocytes, the lectin displayed strong hemagglutination activity down to concentration of 2.3 nM. The recombinant protein is stable and demonstrates a denaturation temperature of 56°C by thermal shift analysis (S2 Fig). Further stabilization of rPVL can be obtained in the presence of GlcNAc-containing ligands like GlcNAcβ1-3Gal or chitobiose, with a Tm of 60°C for both, but not in the presence of non-binding carbohydrate such as galactose and sucrose, confirming the GlcNAc binding activity.

### rPVL is specific for oligosaccharides with terminating GlcNAc residues

**Fig 2:**
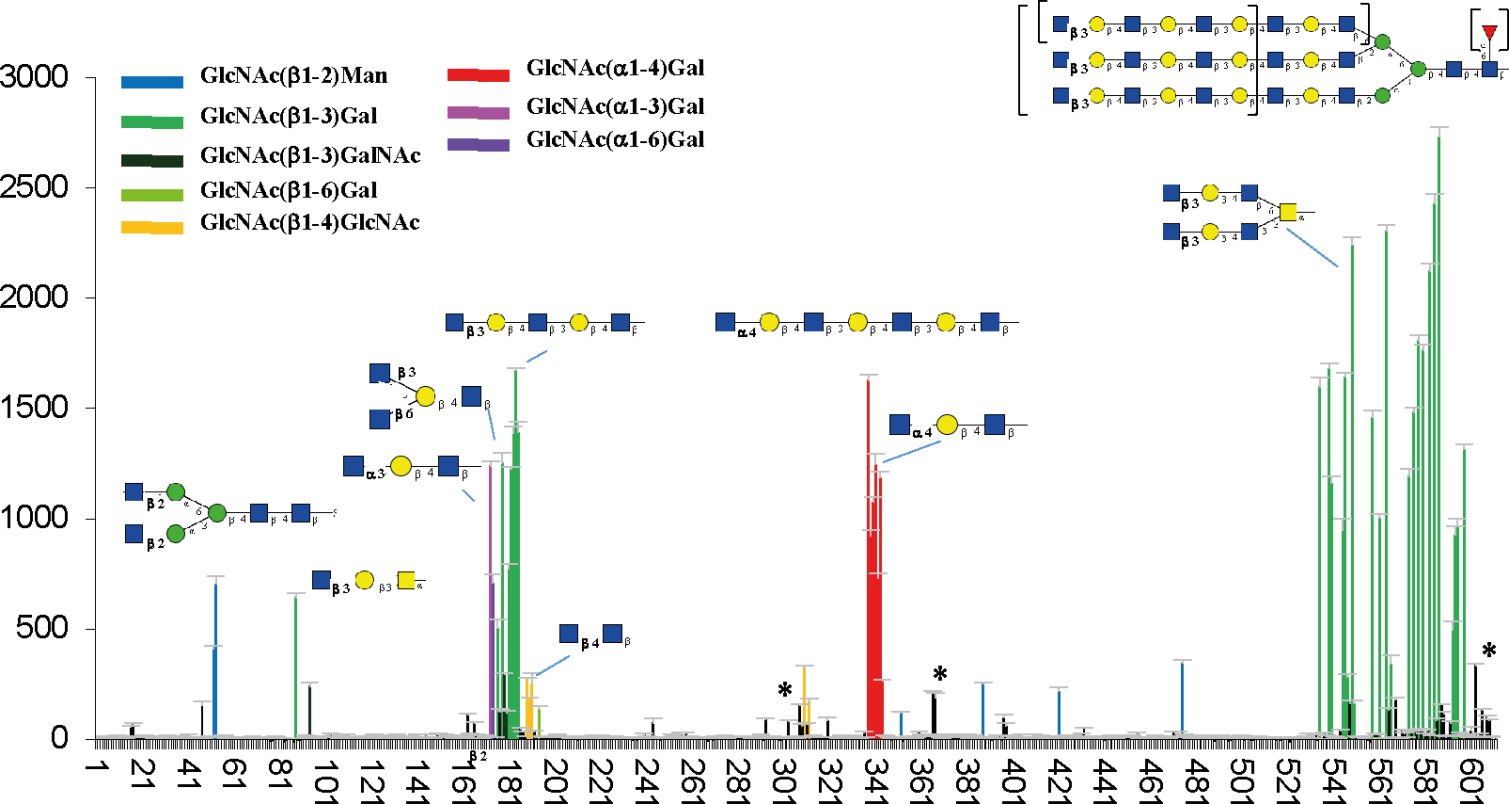
Direct fluorescence measurement obtained with Alexa Fluor 488 labeled rPVL (0.2 μl ml^-1^) on the Mammalian Printed Array version 5.1 from the Consortium for Functional Glycomics. Each hit has been colored according to the terminal disaccharide. Representative oligosaccharides have been schematically displayed. Peaks indicated by a * correspond to minor labeling of sialic-acid terminated oligosaccharides. The full list of oligosaccharides with fluorescence intensities is available from CFG web site (http://www.functionalglycomics.org).

The specificity of recombinant PVL was assayed on the Mammalian Printed Array version 5.1 with 610 glycans, mostly from mammalian origin. The rPVL concentration was varying from 0.2 mg ml^-1^ to 0.2 μg ml^-1^ and the four datasets are available from the CFG site with primscreen_codes 5693, 5695, 5696 and 4697 (http://www.functionalglycomics.org). The fluorescence signal was excellent and the lowest concentration was sufficient for the determination of the strong preference for oligosaccharides carrying a GlcNAc at their non-reducing end (Fig 2).

The lectin bound efficiently to GlcNAc present in two different positions in truncated N-glycans: GlcNAcβ1-2Man on the core pentasaccharides and even more strongly on GlcNAcβ1-3Gal on antenna that are extended by polylactosamine motifs. Because of multivalency effect, the signal was maximal for multiantennary structures (2 to 5 antenna) with lactosamine repeats, the occurrence of which is related to cancer progression [27]. Polylactosamine bi- or multi-antennary N-glycans with terminal GlcNAcβ1-3Gal were bound with fluorescence signals higher than 1500 RU, while the ones with terminal Galβ1-4GlcNAc are in the background noise, below 150 RU. No binding is observed to the GlcNAcβ1-4Man motif present in bisected N-glycans, but the presence of this bisecting GlcNAc does not hinder the binding of PVL to neighboring GlcNAc-terminated antenna. The lectin also bound to mucin motifs that bear βGlcNAc on position 3 and 6 of a GalNAc residue (type 4). It bound only weakly to the chitobiose motif (GlcNAcβ1-4GlcNAc) of chitin. The lectin is not selective for the anomeric configuration of the GlcNAc and several oligosaccharides with terminal αGlcNAc residues were also labeled. Such epitopes are present in human heparan sulfate (GlcNAc α1-4IdoA) and in mucins of gastric glands (GlcNAc α1-4Gal) [5], but also in class III mucin of carcinoma tissues that express a gastric phenotype [28]. The glycan array contains a variety of oligosaccharides with terminal GalNAc but PVL binding was observed only to the disaccharide GalNAcβ1-3GalNAc (#97) with minor intensity.

Since immobilized wild-type PVL was reported to bind to sialylated glycoproteins [29], the binding to oligosaccharides with sialylated structures was checked. Indeed a weak binding may be observed for biantennary N-glycans with terminating Neu5Acα2-3Gal epitopes (#603 in Fig 2). The level of fluorescence was close to the noise level (12% of maxium). Nevertheless, when checking the glycan array data obtained with higher concentration of PVL (0.1 mg ml^-1^), the binding to sialyl-oligosaccharides became clearly visible.

The affinity of rPVL for various GlcNAc-containing oligosaccharides was tested for both free and surface bound proteins using titration microcalorimetry and surface plasmon resonance, respectively (Table 1 and S3 Fig). The affinity for GlcNAc is in the same range as that previously measured by ITC with native PVL isolated from mushroom (Kd ≈200 μM) [13]. Quantitative measurements confirmed a stronger affinity for GlcNAcβ1-3Gal-terminated structures than for GlcNAc and chitooligosaccharides, with dissociation constant of about 70 μM for the trisaccharide lacto-N-triose. A good agreement was observed between values measured by both methods. Only the N-glycan heptasaccharide presenting two terminal GlcNAcβ1-2Man epitopes showed stronger affinity for rPVL in solution (Kd of 34 μM) than on SPR chip. This may be due to the bivalent character of this particular interaction that may be more easily established with the protein in solution than fixed on a chip. The binding of both terminal GlcNAc residues of the biantennary heptasaccharide was confirmed by comparing the stoichiometry of linear GlcNAcβ1-3Gal (five ligands per rPVL) and of biantennary heptasaccharide (two ligands per rPVL). The thermodynamics of binding was also different since the biantennary glycan is the only one to present a favorable entropy of binding (Table 1), that could be explained by the preorientation of the two GlcNAc residues in space.

**Table 1:** Determination of affinity characteristics and thermodynamic contributions for the binding of rPVL with different oligosaccharides.

glycan	SPR	ITC			
	Kd (μM)	Kd (μM)	n	-∆H (kJ/mol)	T∆S (kJ/mol)
GlcNAc	132	124	5.0^b^	41.2	-18.0
GlcNAcβ1-4GlcNAc ^a^	205	122	5.0^b^	36.9	-14.6
GlcNAc(β1-4GlcNAc)_4_	358	n.d.			
GlcNAcβ1-3Gal	103	110	4.9 ± 0.2	42.1	-19.5
GlcNAcβ1-3Galβ1-4Glc (LNT2)	71	108	5.0 ± 0.2	41.7	-19.1
Biantennary N-glycan (**2**) (with terminal GlcNAcβ1-2Man)	179	34.4	1.9 ± 0.6	13.7	11.8
Galβ1-3GlcNAcβ1-3Galβ1-4Glc (LNT)	No binding	n.d.			

^a^ only one experiment by ITC

^b^ stoichiometry value fixed during fitting procedure.

Standard deviation lower than 20% were obtained for ITC experiments.

### Crystal structure of rPVL/disaccharide

**Fig 3:**
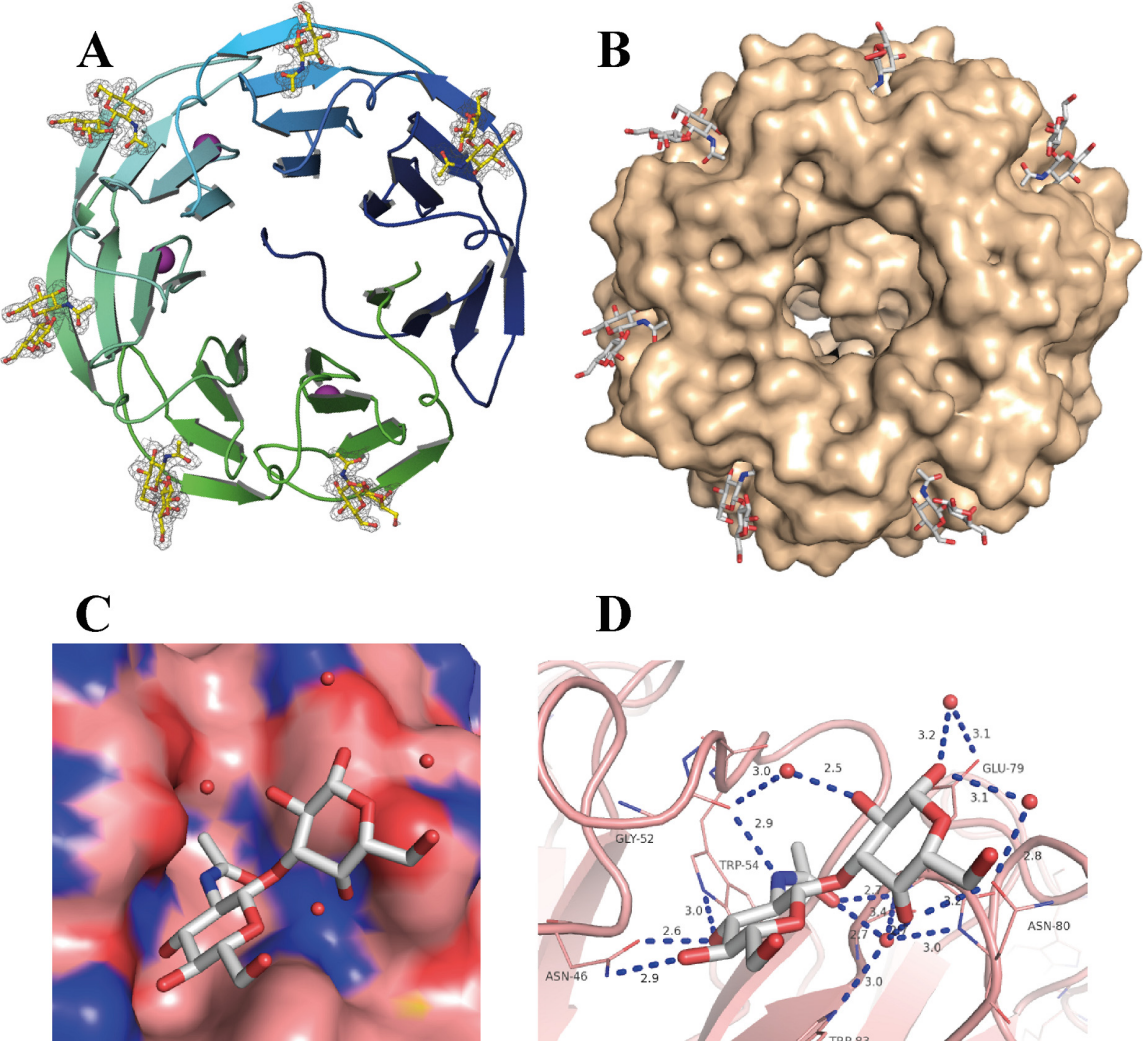
Crystal structure of rPVL/GlcNAcβ1-3Gal complex. A. β-propeller fold of rPVL with the peptide chain represented as ribbon colored from blue (N-terminal) to green (C-terminal). Disaccharides are represented as stick with 2mFo-DFc electron density maps contoured at 1 σ (0.4 ε Å^**-3**^). Calcium ions are represented as pink sphere. B. Same propeller with solvent accessible surface of the protein. C and D, details of the interaction in site 1 with disaccharide and water molecules. Hydrogen bonds are represented as dotted lines in panel D. All graphics have been prepared on chain A using the PyMol software (Schrödinger).

rPVL was crystallized in complex with GlcNAcβ1-3Gal, the disaccharide that caps truncated polylactosamine N-glycans. The structure was solved at 1.95 Ǻ resolution (S1 Table) and displayed the expected seven-bladed β-propeller fold (Fig 3). Two β-propellers are present in the asymmetric unit, but since they are very similar, only chain A is described below. The overall structure of the recombinant protein was very similar to the one described previously for wild-type PVL [13]. The main difference was the presence of an additional calcium ion since rPVL presents calcium in three loops. Clear electron density was observed for the GlcNAcβ1-3Gal in five binding sites while site 2 was occupied only by a GlcNAc residue (Fig 3A and 3B).

In each of the six binding sites, the terminal GlcNAc was in the same orientation as in the PVL/GlcNAc complex, and established the same network of hydrogen bonds. The galactose residue interacted in a shallow area near the GlcNAc binding site (Fig 3C). The interaction of the galactose residue were not very strong, and varied from one site to the other. Nevertheless, one common feature was the involvement of a water molecule that was present in all the sites and bridges the disaccharides (atoms O-acetyl of GlcNAc and O-4 of galactose) with backbone nitrogen atoms of the protein and with side chains of the conserved aromatic rings (Trp or Tyr) that stacked against the GlcNAc surface (Fig 3D). This water molecule played a role in PVL specificity since only the galactose residue presented an axial O-4 hydroxyl in an appropriate position to bind to it.

The limited interaction of the galactose residue with the PVL surface was in agreement with the limited change of affinity that was observed when comparing GlcNAcβ1-3Gal with GlcNAc ligands. Nevertheless, the orientation of the reducing ends on the protein ring was in perfect agreement with the strong binding to glycoconjugate surfaces. Indeed, all galactose reducing ends pointed in the same direction, so that mulitantennary glycans or glycoproteins could easily display a strong multivalency effect

### Several GlcNAc-transferases are upregulated in lung carcinoma

As lung carcinoma have been previously shown to express high levels of GlcNAc-terminated glycans, we analyzed glycosyltransferase expression in transcriptomic data from 27 paired lung adenocarinoma and adjacent “normal” tissues, available in the NCBI database [30]. We found statistically significant variations between cancer and healthy tissues for several glycosyltransferase genes (S2 Table). In particular, expression of the GlcNAc transferases *GcNT3* (one of the GlcNAcTs responsible for the synthesis of the core 2 O-glycan structures) and *B3GNT3* (responsible for the synthesis of the extended core 1 O-glycan structure) were found to be markedly increased in the adenocarcinoma tissues. Those data are compatible with an increase of the GlcNAc terminated structures in lung cancer cells.

### rPVL strongly binds to GlcNAc and Neu5Ac epitopes on human cancer cells

**Fig 4:**
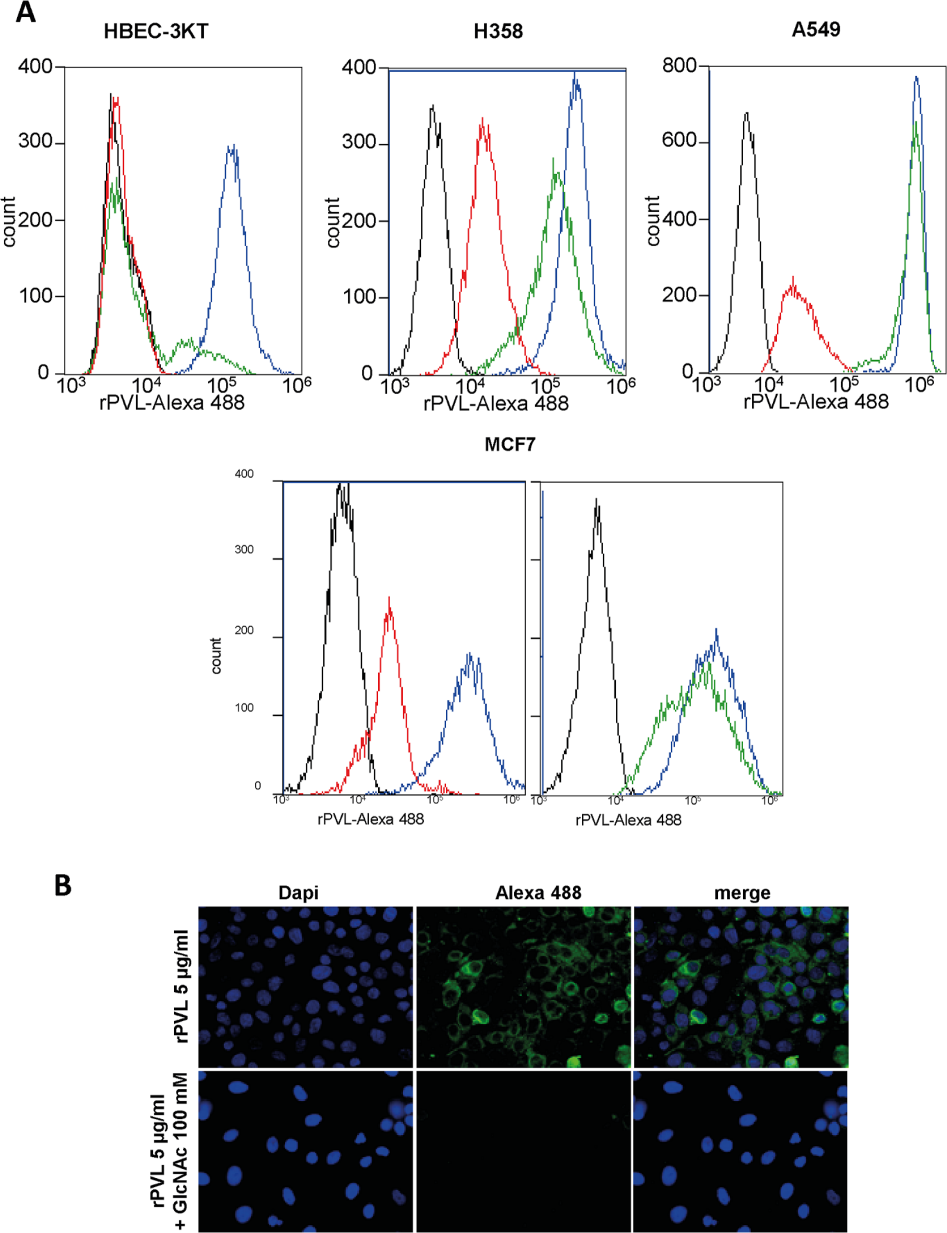
PVL binding of tumor cell lines. A. Flow cytometry histograms show rPVL-Alexa 488 binding to a lung immortalized cell line (HBEC-3KT), two lung tumor cell lines (H358 and A549) as well as on a breast tumor cell line (MCF-7). The x axis indicates fluorescence intensity. The y axis indicates cell number. Black line: untreated control cells; blue line rPVL-Alexa 488 5 μg ml^-1^ for 30 mn; red line: rPVL-alexa 488 5 μg ml^-1^ in the presence of GlcNAc 100 mM; green line: rPVL-Alexa 488 5 μg ml^-1^ after sialidase pretreatment. B. Microscopy images of A549 NSCLC cells treated for 30 min at 37°C with 5 μg ml^-1^ rPVL labeled with Alexa 488 in the presence or absence of 100 mM GlcNAc. Green channel shows rPVL-Alexa 488, blue channel shows nuclei labeled with DAPI staining.

To examine the capacity of rPVL to recognize cells derived from human cancer tissues, lectin staining of several cell lines was performed and quantitatively analyzed using flow cytometry. rPVL strongly bound to cancer cells from various origins, including lung (Fig 4 and S4 Fig and S3 Table), breast, prostate, ovary and colon carcinomas, as well as melanomas.

In the presence of 100 mM GlcNAc, the binding of rPVL to the cell lines was strongly decreased, indicating specific involvement of the carbohydrate binding sites (Fig 4). Interestingly, complete inhibition was achieved only for the HBEC-3KT cell line which is an immortalized normal lung cell line, suggesting that rPVL’s binding sites on this cell line may be of lower affinity than on the cancer cell lines. Since the glycan microarray showed some binding to Neu5Ac containing glycans, sialidase treatment was performed in order to determine if Neu5Ac-terminated cell surface glycans could be recognized by the lectin. As shown on Fig 4A, following sialidase treatment, cell staining was almost completely lost for the HBEC-3KT cell line, but much less so for other cell lines such as MCF-7 or A549. This result indicated that Neu5Ac-terminated glycans represent the major rPVL ligands on the HBEC-3KT normal lung cell line, whereas GlcNAc-terminated structures may be involved on the cancerous cell lines.

**Fig 5:**
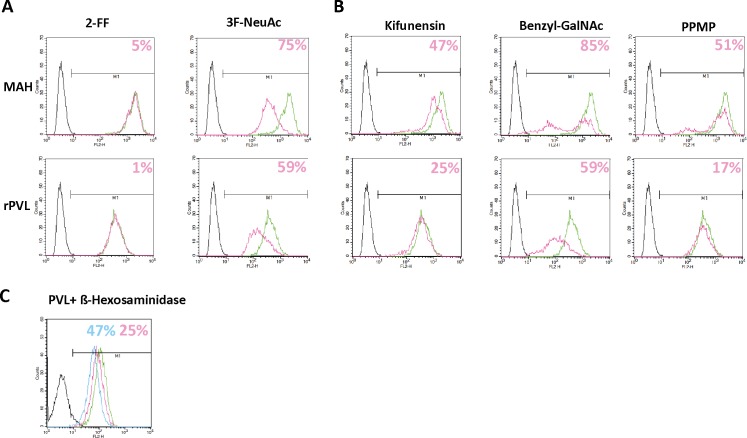
PVL binding in the presence of glycosylation inhibitors or after treatment with glycosidases. A. Biotinylated rPVL (1 μg ml^**-1**^) or MAH (5 μg ml^**-1**^) were incubated with A549 cells grown for 4 days in presence of DMSO (green line) or of either 400 μM 2-Fluoro-Fucose or 100 μM Fluoro-Neu5Ac (pink line). Lectin binding was revealed by PE conjugated Streptavidin. Percentage of inhibitions are indicated according to the mean fluorescence intensities. B. Similar experiment than in A but with A549 cells grown in the presence of 5 μM Kifunensine for 4 days or in the presence of either 6 mM Benzyl-GalNAc or 10 μM PPMP for 48h. C. Similar experiment than in A and B but with cells treated with 2.5 U (pink line) or 12.5 U ß-D-N-acetyl-hexosaminidase (blue line).

To confirm involvement of GlcNAc-terminated glycans on cancer cells such as A549 that showed little decrease of rPVL binding following sialidase treatment, cells were grown in the presence of 3F-Neu5Ac, an inhibitor of sialyltransferases. As a control for specificity, 2F-fucose, an inhibitor of fucosyltransferases was used (Fig 5A). The latter compound did not affect binding of either the Neu5Ac-specific lectin MAH or of rPVL. By contrast, 3F-Neu5Ac, decreased MAH binding by 75%, as expected, but also rPVL binding by 59%, indicating that a significant level of rPVL binding to these cells is due to recognition of Neu5Ac epitopes. Treatment of A549 cells by an exo ß-D-N-acetyl—hexosaminidase also resulted in a major decrease of rPVL binding in a dose-dependent manner, indicating that GlcNAc-terminated glycans are also recognized by rPVL on this cell line. To further delineate which type of glycans are involved, A549 cells were treated with kifunensin, an inhibitor of N-glycan maturation, with benzyl-GalNAc, an inhibitor of O-glycosylation and with PPMP, an inhibitor of glycolipid synthesis (Fig 5B). Treatment by all three inhibitors resulted in a decrease of the MAH Neu5Ac-specific lectin, indicating that Neu5Ac termini are present on these three major types of glycans. Likewise, each inhibitor decreased rPVL binding to some extent. Yet, inhibition of rPVL binding was much clearer following benzyl-GalNAc treatment than following the other treatments. Collectively, these data indicate that both Neu5Ac and GlcNAc present on O-glycans constitute the major ligands for rPVL binding on the A549 lung carcinoma cell line.

### Labeling of healthy tissues by rPVL

**Fig 6:**
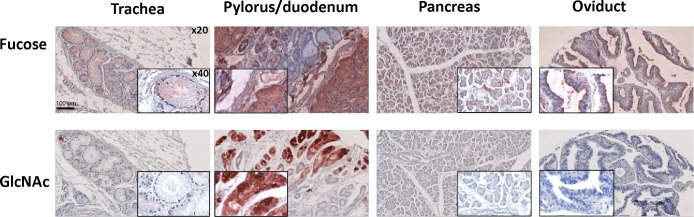
PVL staining of healthy tissues. A tissue microarray (TMA) comprising healthy tissues from respiratory, digestive and genital origin was stained with 0.7 μg ml^**-1**^ rPVL-biot in presence of 0.1 M Fucose or 0.1 M GlcNAc followed by Streptavidin-HRP. AEC was used as a peroxidase substrate to reveal the PVL staining and counterstaining was performed using hematoxylin. Slides were imaged using a NanoZoomer slide scanner with a 20x magnification. 40x digital magnifications are also shown as insets.

Labeling of healthy tissues by biotinylated rPVL was performed in the presence of either fucose or GlcNAc to delineate specific binding. Most tissues were either not stained or only weakly stained by the labeled lectin. When present, staining was mainly restricted to epithelial cells or occasionally to some macrophages in the lung parenchyma and was inhibited by the presence of 100 mM GlcNAc (Fig 6 and S4 Table). owever, a strong binding was observed on the glandular compartment of the stomach and small intestine. In this case, only a partial inhibition could be obtained in the presence 100 mM GlcNAc, consistent with the presence of mucin presenting terminal α-linked GlcNAc in these tissues, as previously described [5, 31].

### Labeling of cancer tissues by rPVL

**Fig 7:**
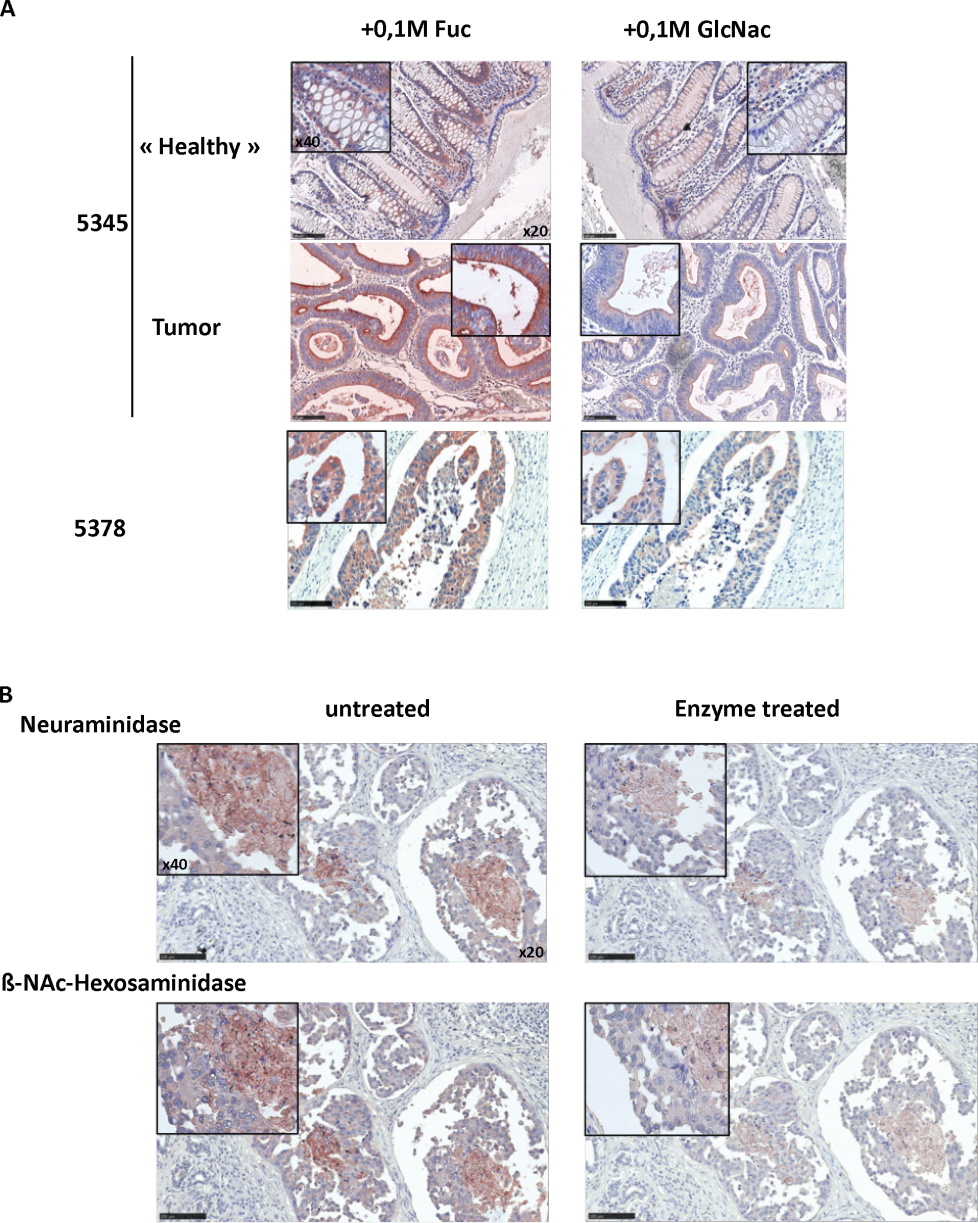
PVL binds to tumor tissue with a mixed Neu5Ac and GlcNAc specificity. A. Sections from ethanol-fixed colon carcinoma and adjacent healthy tissue from 2 different patients (# 5345 and 5378) were stained with 1 μg ml^**-1**^ rPVL-biot in presence of 0.1 M Fucose or 0.1 M GlcNAc followed by Streptavidin-HRP. AEC was used as a peroxidase substrate to reveal the rPVL staining and counterstaining was performed using hematoxylin. Slides were imaged using a NanoZoomer slide scanner with a 20x magnification.40x digital magnifications are also shown as insets. B. Canine breast tumor sections (formalin fixed) were treated or not with glycosidases and then stained with 2 μg ml^**-1**^ rPVL-biot and imaged as in A.

Since rPVL labeled various cancer cell lines, a histochemical staining of healthy and cancer tissues was performed. As shown on Fig 7A a strong labeling, inhibited in the presence of GlcNAc, was observed on colon carcinomas, whilst a weak labeling was only found on the adjacent normal tissue. To determine if the staining of carcinoma cells was dependent on either Neu5Ac or GlcNAc-terminated glycans, tissue sections of canine breast carcinoma were treated with either sialidase or exo-ß-D-N-acetyl-hexosaminidase prior to incubation with the biotinylated lectin (Fig 7B). Canine breast carcinoma sections were used since they were more easily available than human breast carcinoma sections and since the tumors are remarkably homologous to their human counterparts [32]. Both enzyme treatments led to a clear reduction of the staining, indicating that similar to what was observed on cell lines, both Neu5Ac and GlcNAc were involved in the lectin staining.

**Fig 8:**
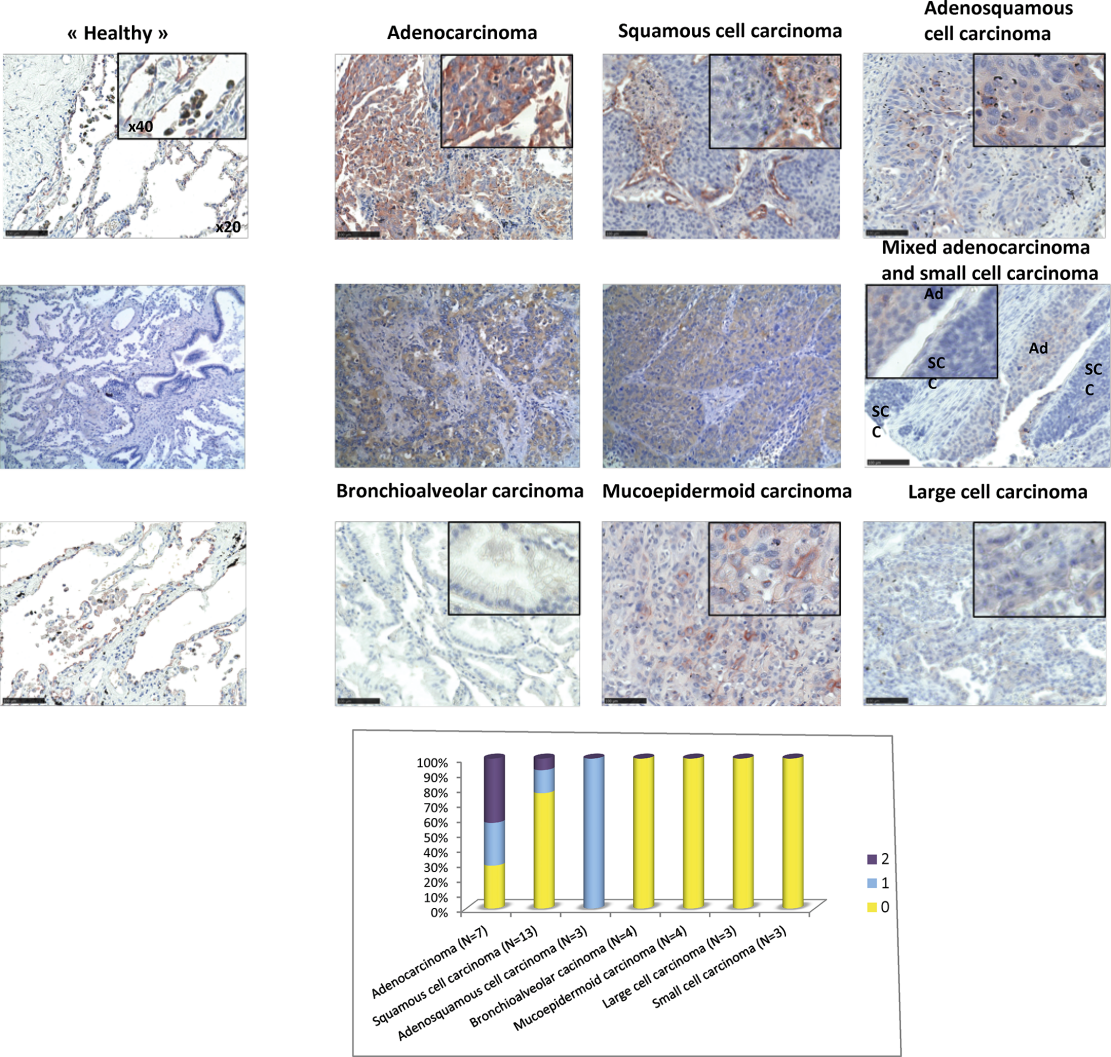
PVL staining of Lung tumoral tissues. A. A lung tumor TMA (formalin-fixed) was stained with 2 μg ml^**-1**^ rPVL-biot in presence of 0.1 M fucose followed by Streptavidin-HRP. AEC was used as a peroxidase substrate to reveal the PVL staining and counterstaining was performed using hematoxylin. Slides were imaged using a NanoZoomer slide scanner with a 20x magnification. 40x digital magnifications are also shown as insets. (Ad = adenocarcinoma; SCC = small cell carcinoma) Representative images of the different histological tumor types are shown B. A score from 0 to 3 was attributed to each tumor, based on the percentage of tumor cell stained and the color intensity and the staining level distribution according to tumor histological subtypes has been represented as an histogram.

We next analyzed staining of various types of lung and breast carcinomas with rPVL. A clear labeling was observed on a proportion of lung squamous cell carcinomas, and lung adenocarcinomas (Fig 8 and S5 Fig. The staining of cancer cells with rPVL was systematically higher than that of the normal surrounding cells in each positive tumor sample. By contrast, none of the tested bronchioalveolar, mucoepidermoid, large cell or small cell carcinomas that we tested was labeled, suggesting that the lectin could distinguish between distinct types of lung carcinomas.

**Fig 9:**
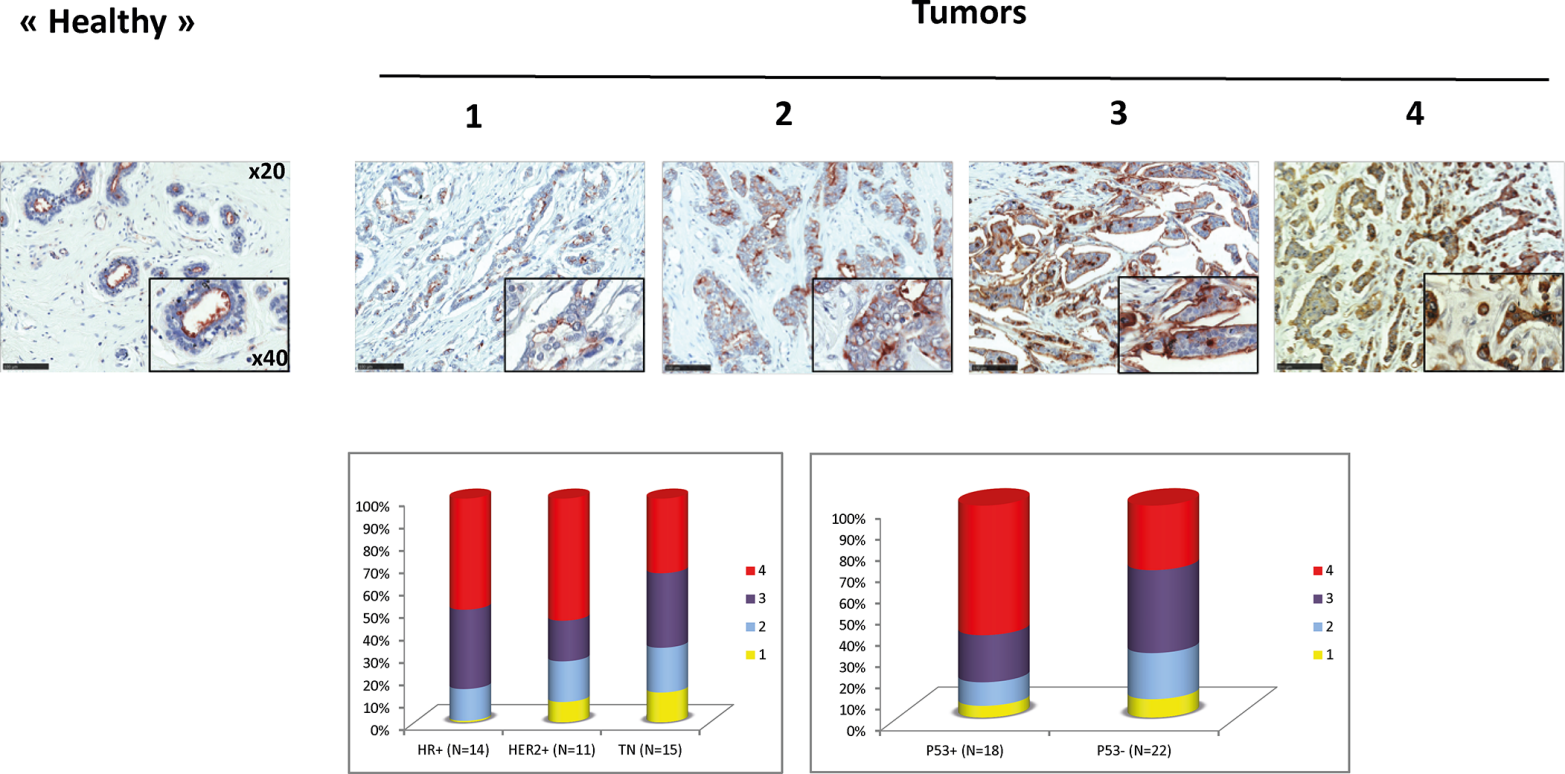
PVL staining of breast tumoral tissues. A. A Breast tumor TMA (formalin-fixed) was stained and imaged as described in Fig 8A. Tumors were all positive with a vast majority of cancer cells stained. Labeling intensity was estimated on a scale ranging from 1 to 4. Representative examples of tumors of each staining level are shown. B. Staining level distributions according to tumor molecular characteristics. (HR = Hormone Receptors, TN = Triple Negative).

When biotinylated rPVL was applied to breast tumor sections (Fig 9), the majority of samples appeared positive, although labeling intensity ranged from moderate to very strong. By contrast, in normal breast tissue adjacent to the tumors, only weak staining at the apical surface of canular epithelial cells could be occasionally observed. With the limited number of samples available, no significant difference in staining intensity could be observed between tumors with different HER2, hormone receptors or p53 status.

## Discussion and Conclusion

The recombinant form of PVL has been produced and characterized, and its binding specificity is different from that of other GlcNAc-binding lectins according to the specificity patterns available from the Consortium for Functional Glycomics. Wheat germ agglutinin (WGA) and tomato lectin (SLT) recognize terminal GlcNAc, but also bind to internal GlcNAc and to chitooligosaccharides. The legume lectins from *Ulex europeus* (UEA-II) and *Griffonia (Bandereia) simplicifolia* (GSL-II) display high specificity to truncated polylactosamine-type N-glycans only. F17G, a lectin present on the pili of some *E*. *coli* strains, has also recently been shown to specifically recognize terminal GlcNAcß1-3Gal motifs, although with a rather low affinity [33]. The lectin with the closest specificity pattern to PVL is AAL-2 from the mushroom *Agrocybe aegerita*. This related protein (60% identity) has also been produced recombinantly and demonstrated to bind to hepatoma cells and to induce apoptosis, although no information was provided on the binding event. [14].

The present work opens the route for further investigations. Although each of the six binding sites of PVL is highly specific for terminal GlcNAc, the multivalent lectin displays weak avidity for Neu5Ac-decorated surfaces that may result in weak non-specific labeling of some tissues. Since we aim at targeting specifically GlcNAc-terminated residues, we demonstrated that the use of sialidase treatment and/or competition with low amounts of GlcNAc suppressed the non-specific binding. Nevertheless, the structural knowledge of the binding sites should allow for engineering of modified lectins with stronger selectivity.

Because of its capacity to bind to nonreducing terminal GlcNAc, rPVL is a marker of interest for cancer cells, as demonstrated here, However, rPVL could also have diagnostic applications for other diseases. For example, an elderly dementia, distinct from Alzheimer disease, is related to the presence of an agalacto-N-glycan on one isoform of transferrin [34]. Also agalacto-IgGs are present in the serum of patients with rheumatoid arthritis [35] and were previously detected with PVL purified from fungi [36, 37] or by use of glycosyltransferase assays [38]. Some fast replicating viruses such as Ebola virus [39] and SARS coronavirus [40] were also demonstrated to carry truncated N-glycans with exposed GlcNAc residues.

It was previously proposed that the truncated N-glycans observed on Ebola virus may be due to their fast replication resulting in overloading of the processing machinery [39]. Such a hypothesis could also stand for anarchically developing cancer cells for which glycosylation could be poorly processed. However, we could demonstrate that the alterations of surface glycosylation on cancer cells are correlated to modifications of glycosyltransferase expression levels (S2 Table). Specific glycosylation changes may therefore occur in these cells and further work is needed to investigate the mechanims underlying such changes of glycosylation associated with cancer.

As demonstrated in the present work, rPVL histochemical staining could be a powerful tool to help the pathological analysis of human non-small cell lung cancers, as well as breast and colon carcinomas. However, this will require further validation on larger cohorts of patients. More particularly, larger scale analysis would permit to establish correlation with prognosis in different subgroups of tumors. Other lectins were proven to be of interest, such as the GalNAcβ1-3/4GlcNAc specific lectin from *Wisteria japonica* seeds that binds strongly to lung squamous cell carcinoma [41]. Therefore, the further development of lectin arrays is very promising for a better detection of glycan alterations for diagnostic purposes.

## Supporting Information

S1 Fig ^1^H NMR of heptasaccharide azide 2. ^1^H-NMR (360 MHz, D_2_O): δ = 5.03 (d, *J*_1,2_ < 1 Hz, 1H, H-1^4^), 4.83 (d, *J*_1,2_ < 1 Hz, 1H, H-1^4´^), 4.68–4.64 (m, 2H, H-1^1^, H-1^3^), 4.53 (d, *J*_1,2_ = 7.7 Hz, 1H, H-1^2^), 4.47 (d, *J*_1,2_ = 8.3 Hz, 2H, H-1^5^, H-1^5’^), 4.18–4.15 (m, 1H, H-2^3^), 4.12–4.09 (m, 1H, H-2^4^), 4.04–4.01 (m, 1H, H-2^4’^), 1.99 (s, 3H, NAc), 1.97 (s, 9H, NAc).
(PDF)

S2 Fig Thermal stability of rPVL. (A) Evolution of fluorescence of Sypro Orange binding to denaturing rPVL at 0.5 mg ml^-1^ and 0.1 m. ^-1^ (*) with glycan ligands. (B) Denaturation curve derivatives. RFU: Relative Fluorescence Units.
(PDF)

S3 Fig ITC data. Raw ITC data (top) obtained by injections of oligosaccharides in a solution of rPVL and the respective integrated titration curve (bottom). Left, 7 mM of GlcNAcβ1-3Gal into 0.06 mM of rPVL. Right, 9 mM of Heptasacharide in 0.1 mM of rPVL.
(PDF)

S4 Fig Microscopy images of H358 NSCLC cells untreated or treated for 30 min at 37°C with increasing concentrations of rPVL labelled with Alexa 488. Green channel shows rPVL-Alexa 488, blue channel shows nuclei labelled with DAPI staining.
(PDF)

S5 Fig rPVL labelled with biotin with paraffin-embedded tissue sections of normal human lung (A and B) or human lung adenocarcinoma (C-F), using the streptavidin-peroxidase technique. Sections are counterstained with hematoxylin. Left column (A, C, and E): control without rPVL. Right column (B, D, and F): 5 μg ml^-1^ rPVL-treated sections. Original magnifications: x4 (A, C, D); x20 (B, E, F).
(PDF)

S1 Table Data Collection and Refinement Statistics for rPVL- GlcNAcβ1-3Gal complex structure. (PDF)

S2 Table Expression of glycosyltransferases in lung cancer. Glycosyltransferases expression levels were extracted from a transcriptome analysis of 27 lung adenocarinoma and adjacent normal tissues, available in the NCBI database (Su L et al., *BMC Genomics* 2007; GEO accession: GSE 7670), thanks to the BioGPS programme (http://biogps.org). A) Summary of the glycosyltransferase expression trends in tumor versus normal tissues. A 2-sided paired t-Test was used to check for statistical difference between the normal and cancer tissue expression (note that the given p-values have not been corrected for multiple testing). B) Raw data. The left column corresponds to the patient identification numbers as recorded in the dataset. Data for B3GNT 6, 7, 8, 9 and ST3GAL 3 were not available in this dataset. Note that B3GalT3 mainly shows a GalNAc transferase activity in the Gb4Cer globoside synthesis (P antigen).
(PDF)

S3 Table rPVL binding on various cancer cells. Binding of rPVL was determined by flow cytometry on cancer cell lines from various histological origins as described in Material and Methods. ND: not determined.
(PDF)

S4 Table PVL staining of healthy tissues. A TMA prepared with tissues from various origins coming from 10 different donors was stained with rPVL-biot at 0,7 μg/ml as such or in presence of 0,1 M of fucose or N-acetyglucosamine. Digestive tissues showed the strongest staining; some of it could not be completely removed even in presence of an excess of free GlcNAc.
(PDF)
