# Supplementary material for: A Recombinant Fungal Lectin for Labeling Truncated Glycans on Human Cancer Cells
Source: PLoS One. 2015 Jun 4;10(6):e0128190. doi: 10.1371/journal.pone.0128190 (PMC4456360; doi:10.1371/journal.pone.0128190)
Supplement: S1 Fig — 1H-NMR (360 MHz, D2O): δ = 5.03 (d, J 1,2 < 1 Hz, 1H, H-14), 4.83 (d, J 1,2 < 1 Hz, 1H, H-14´), 4.68–4.64 (m, 2H, H-11, H-13), 4.53 (d, J 1,2 = 7.7 Hz, 1H, H-12), 4.47 (d, J 1,2 = 8.3 Hz, 2H, H-15, H-15’), 4.18–4.15 (m, 1H, H-23), 4.12–4.09 (m, 1H, H-24), 4.04–4.01 (m, 1H, H-24’), 1.99 (s, 3H, NAc), 1.97 (s, 9H, NAc). (PDF) [file pone.0128190.s001.pdf]

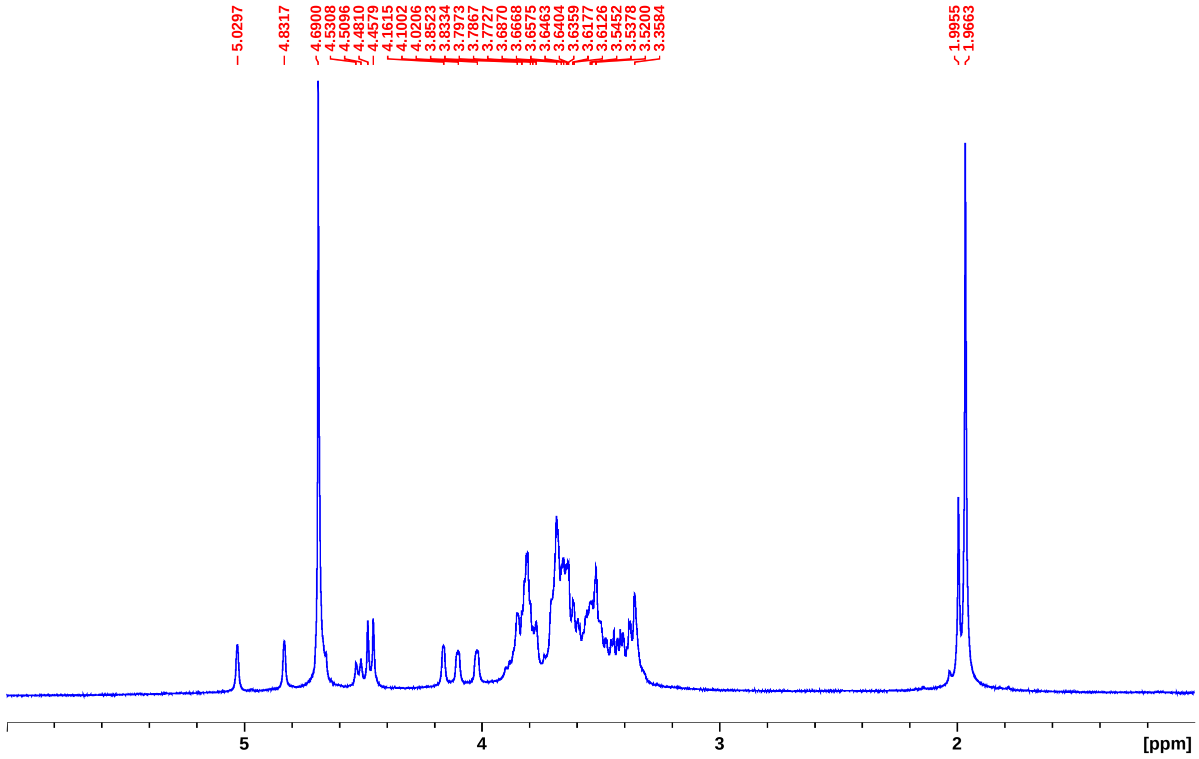

Figure 1S: ^{1}H NMR of heptasaccharide azide **2**. ^{1}H-NMR (360 MHz, D_2O): δ = 5.03 (d, $J_{1,2}$ < 1 Hz, 1H, H-1^{4}), 4.83 (d, $J_{1,2}$ < 1 Hz, 1H, H-$1^{4'}$), 4.68-4.64 (m, 2H, H-1^{1}, H-1^{3}), 4.53 (d, $J_{1,2}$ = 7.7 Hz, 1H, H-1^{2}), 4.47 (d, $J_{1,2}$ = 8.3 Hz, 2H, H-1^{5}, H-$1^{5'}$), 4.18-4.15 (m, 1H, H-2^{3}), 4.12-4.09 (m, 1H, H-2^{4}), 4.04-4.01 (m, 1H, H-$2^{4'}$), 1.99 (s, 3H, NAc), 1.97 (s, 9H, NAc).
