# Supplementary material for: A Recombinant Fungal Lectin for Labeling Truncated Glycans on Human Cancer Cells
Source: PLoS One. 2015 Jun 4;10(6):e0128190. doi: 10.1371/journal.pone.0128190 (PMC4456360; doi:10.1371/journal.pone.0128190)
Supplement: S2 Fig — (A) Evolution of fluorescence of Sypro Orange binding to denaturing rPVL at 0.5 mg ml-1 and 0.1 m. -1 (*) with glycan ligands. (B) Denaturation curve derivatives. RFU: Relative Fluorescence Units. (PDF) [file pone.0128190.s002.pdf]

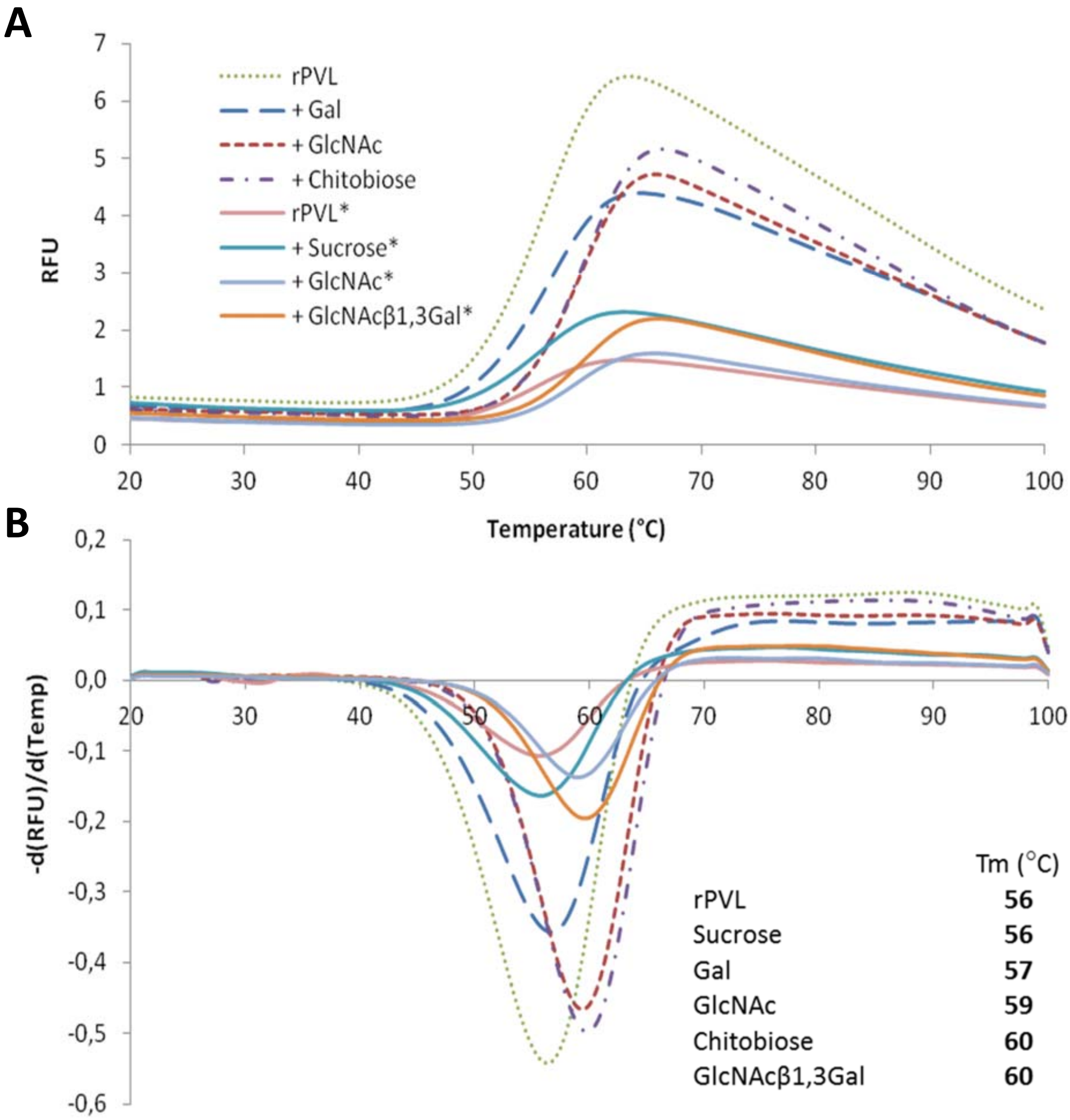

Figure S2: Thermal stability of rPVL. (A) Evolution of fluorescence of Sypro Orange binding to denaturing rPVL at 0.5 mg ml^{-1} and 0.1 m $.ml^{-1}$ (*) with glycan ligands. (B) Denaturation curve derivatives. RFU: Relative Fluorescence Units.
