# Supplementary material for: A Recombinant Fungal Lectin for Labeling Truncated Glycans on Human Cancer Cells
Source: PLoS One. 2015 Jun 4;10(6):e0128190. doi: 10.1371/journal.pone.0128190 (PMC4456360; doi:10.1371/journal.pone.0128190)
Supplement: S3 Fig — Raw ITC data (top) obtained by injections of oligosaccharides in a solution of rPVL and the respective integrated titration curve (bottom). Left, 7 mM of GlcNAcβ1-3Gal into 0.06 mM of rPVL. Right, 9 mM of Heptasacharide in 0.1 mM of rPVL. (PDF) [file pone.0128190.s003.pdf]

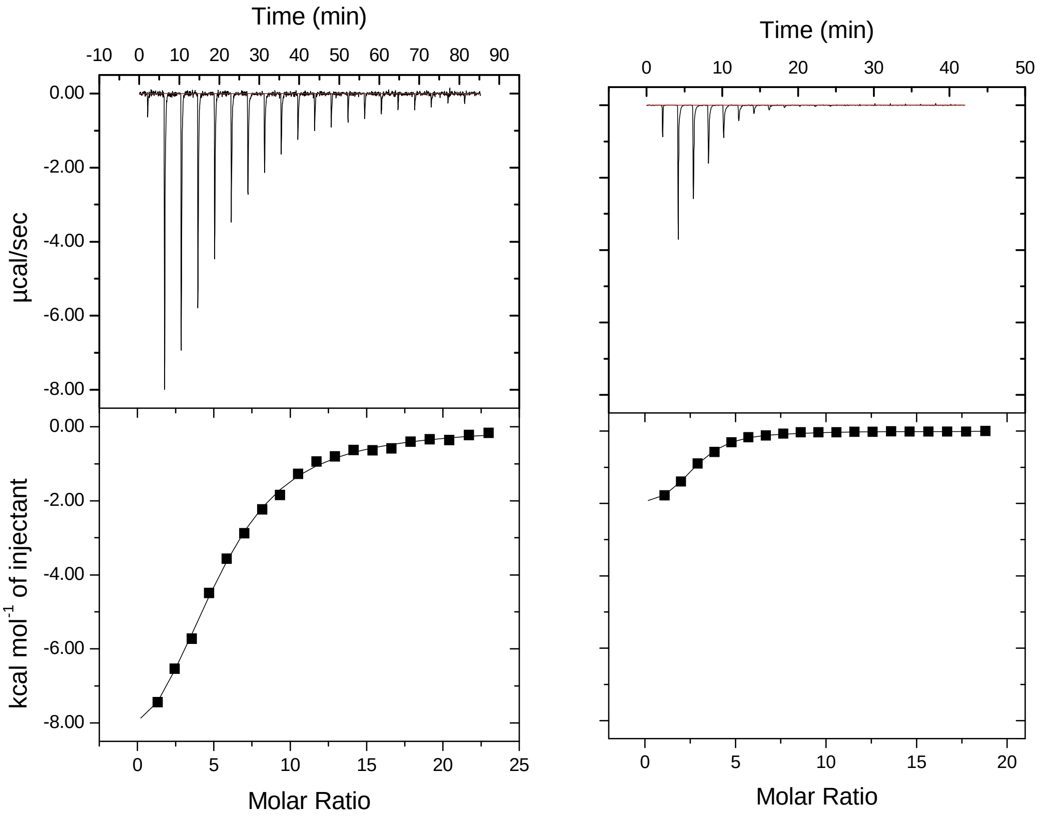

Figure S3: Raw ITC data (top) obtained by injections of oligosaccharides in a solution of rPVL and the respective integrated titration curve (bottom). Left, 7 mM of GlcNAcβ1-3Gal into 0.06 mM of rPVL. Right, 9 mM of Heptasacharide in 0.1 mM of rPV
