# Supplementary material for: A Recombinant Fungal Lectin for Labeling Truncated Glycans on Human Cancer Cells
Source: PLoS One. 2015 Jun 4;10(6):e0128190. doi: 10.1371/journal.pone.0128190 (PMC4456360; doi:10.1371/journal.pone.0128190)
Supplement: S4 Fig — Green channel shows rPVL-Alexa 488, blue channel shows nuclei labelled with DAPI staining. (PDF) [file pone.0128190.s004.pdf]

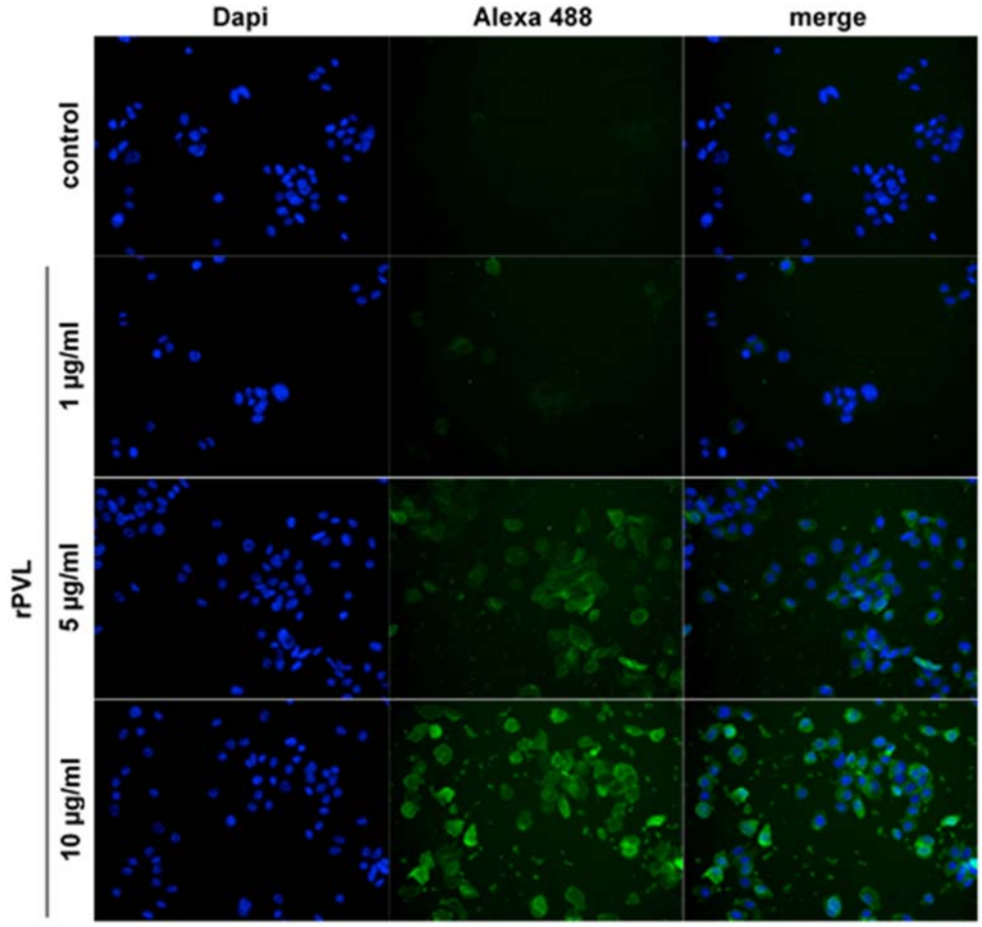

Figure S4: Microscopy images of H358 NSCLC cells untreated or treated for 30 min at 37°C with increasing concentrations of rPVL labelled with Alexa 488. Green channel shows rPVL-Alexa 488, blue channel shows nuclei labelled with DAPI staining.
