# Supplementary material for: A Recombinant Fungal Lectin for Labeling Truncated Glycans on Human Cancer Cells
Source: PLoS One. 2015 Jun 4;10(6):e0128190. doi: 10.1371/journal.pone.0128190 (PMC4456360; doi:10.1371/journal.pone.0128190)
Supplement: S5 Fig — Sections are counterstained with hematoxylin. Left column (A, C, and E): control without rPVL. Right column (B, D, and F): 5 μg ml-1 rPVL-treated sections. Original magnifications: x4 (A, C, D); x20 (B, E, F). (PDF) [file pone.0128190.s005.pdf]

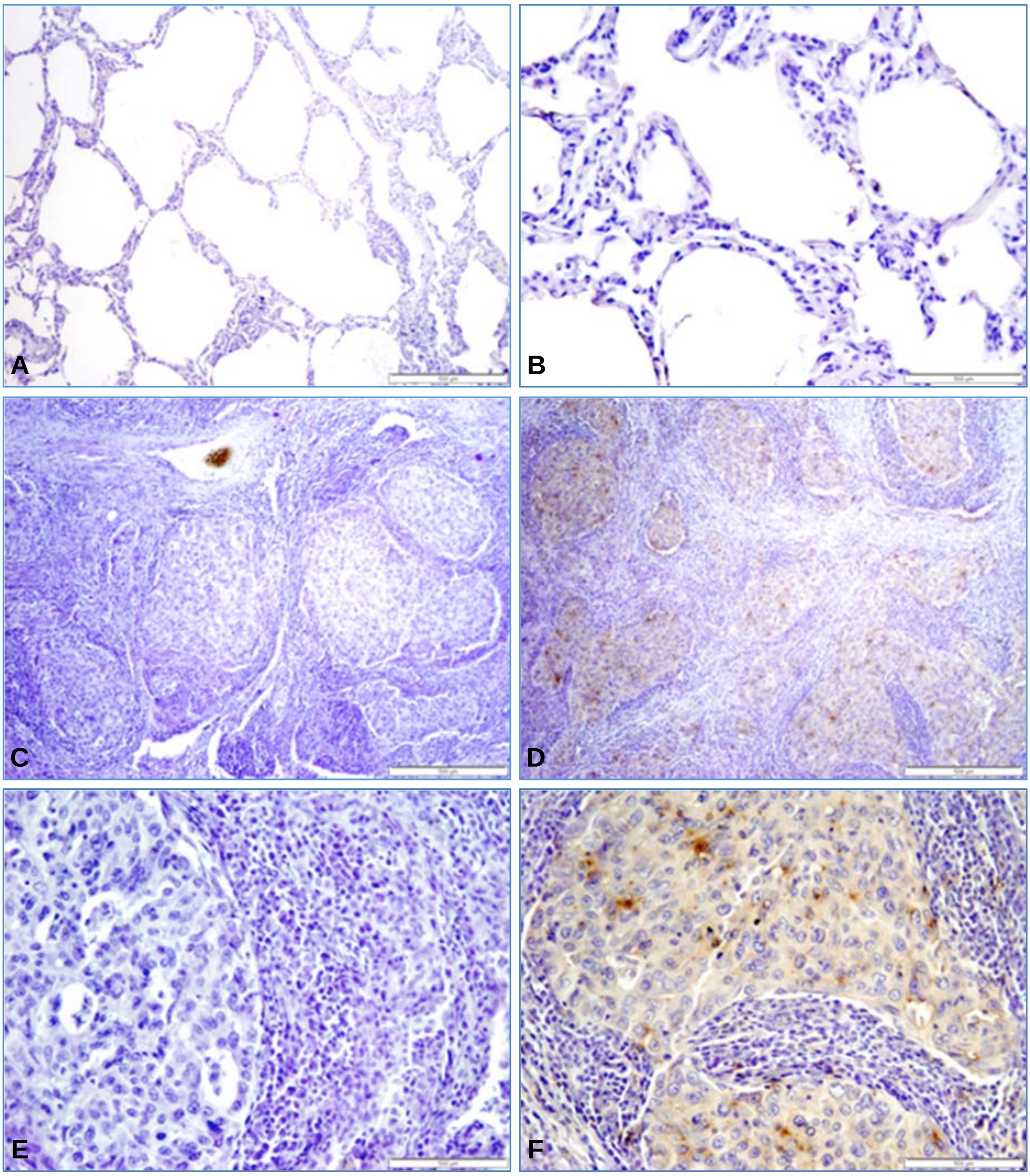

Figure S5: rPVL labelled with biotin with paraffin-embedded tissue sections of normal human lung (A and B) or human lung adenocarcinoma (C-F), using the streptavidin-peroxidase technique. Sections are counterstained with hematoxylin. Left column (A, C, and E): control without rPVL. Right column (B, D, and F): 5 µg ml^{-1} rPVL-treated sections. Original magnifications: x4 (A, C, D); x20 (B, E, F).
