# Supplementary material for: A Recombinant Fungal Lectin for Labeling Truncated Glycans on Human Cancer Cells
Source: PLoS One. 2015 Jun 4;10(6):e0128190. doi: 10.1371/journal.pone.0128190 (PMC4456360; doi:10.1371/journal.pone.0128190)
Supplement: S1 Table — (PDF) [file pone.0128190.s006.pdf]

Table S1: Data Collection and Refinement Statistics for rPVL- GlcNAcβ1-3Gal complex structure

Data		
Beamline (wavelength, Å)	BM30A/0.9205	
Spacegroup	$P2_12_12_1$	
Unit cell dimensions, a, b,c Å	54.81 97.47 158.65	
Resolution (outer shell), Å	30.0-1.95 (2.00-1.95)	
Measured/ Unique reflections	327526/62754	
Average multiplicity	5.2 (4.1)	
R_{merge}	0.101 (0.396)	
R_{pim}	0.073 (0.307)	
Completeness (%)	99.8 (98.3)	
Mean I / σI	11.8 (3.1)	
CC1/2	0.996 (0.848)	
Wilson B	11.3	
Refinement		
R_{cryst}/ R_{free}	19.0/ 23.7	
nb reflections/free reflections	59602/3069	
R_{msd} bonds, Å	0.016	
R_{msd} angles,°	1.69	
Rmsd chiral, Å^3	0.10	
Atoms (chain)	A	B
Protein	3033	3057
Bfac Å^2	16.0	17.9
Sugars	160	145
Bfac, Å^2	21.0	24.2
Calcium	3	3
Bfac, Å^2	22.5	26.6
Water molecules	453	397
Bfac, Å^2	23.3	24.4
Ramachandran (Molprobity)	Allowed region: 100%<br>Favoured region: 98.1%<br>Outliers : 0	
PDBcode	4UP4	
