# Supplementary material for: A Recombinant Fungal Lectin for Labeling Truncated Glycans on Human Cancer Cells
Source: PLoS One. 2015 Jun 4;10(6):e0128190. doi: 10.1371/journal.pone.0128190 (PMC4456360; doi:10.1371/journal.pone.0128190)
Supplement: S2 Table — Glycosyltransferases expression levels were extracted from a transcriptome analysis of 27 lung adenocarinoma and adjacent normal tissues, available in the NCBI database (Su L et al., BMC Genomics 2007; GEO accession: GSE 7670), thanks to the BioGPS programme (http://biogps.org). A) Summary of the glycosyltransferase expression trends in tumor versus normal tissues. A 2-sided paired t-Test was used to check for statistical difference between the normal and cancer tissue expression (note that the given p-values have not been corrected for multiple testing). B) Raw data. The left column corresponds to the patient identification numbers as recorded in the dataset. Data for B3GNT 6, 7, 8, 9 and ST3GAL 3 were not available in this dataset. Note that B3GalT3 mainly shows a GalNAc transferase activity in the Gb4Cer globoside synthesis (P antigen). (PDF) [file pone.0128190.s007.pdf]

Table S2: Expression of glycosyltransferases in lung cancer. Glycosyltransferases expression levels were extracted from a transcriptome analysis of 27 lung adenocarinoma and adjacent normal tissues, available in the NCBI database (Su L et al., *BMC Genomics* 2007; GEO accession: GSE 7670), thanks to the BioGPS programme (http://biogps.org). A) Summary of the glycosyltransferase expression trends in tumor versus normal tissues. A 2-sided paired t-Test was used to check for statistical difference between the normal and cancer tissue expression (note that the given p-values have not been corrected for multiple testing).

Enzyme	comment		Tumor /Normal ratio	P value (Paired t-Test)
**β1-6GlcNAcT**	*Core 2 synthases*	GcNT1	Increased	0.05
		GcNT3	Increased	0.001
		GcNT4	No overall trend	0.4
	I antigen synthase	GcNT2	Increased	0.05
**β1-3GlcNAcT**		B3GNT1	Decreased	0.0008
		B3GNT2	Decreased	0.015
		B3GNT3	Increased	0.00003
		B3GNT4	Increased	0.002
**β1-4GlcNAcT**	*Bisecting GlcNAc*	GNT3	No overall trend	0.9
**β1-2GlcNAcT**		POMGNT1	No overall trend	0.3
**α1-4GlcNAcT**		A4GNT	No overall trend	0.4
**β1-3GalT**		B3GaLT1	No overall trend	1
		B3GaLT2	Decreased	0.001
	(β3GalNT1)	B3GaLT3	Decreased	0.00006
	gangliosides	B3GaLT4	No overall trend	0.5
	O-glycans	B3GaLT5	No overall trend	0.2
**β1-4GalT**		B4GaLT1	No overall trend	0.08
		B4GaLT2	Increased	0.00005
		B4GaLT3	Increased	0.00004
	glycolipids	B4GaLT4	No overall trend	0.06
		B4GaLT5	No overall trend	0.4
		B4GaLT5	No overall trend	0.35
	Proteoglycan	B4GaLT5	Increased	0.0001
**α2-3ST**		ST3GaL1	No overall trend	0.2
		ST3GaL2	No overall trend	0.09
	Sialyl Lewis synthases	ST3GaL4	No overall trend	0.1
		ST3GaL6	No overall trend	1
	GM2 synthase	ST3GaL5	No overall trend	0.1

Table S2 (continuing)

B) Raw data. The left column corresponds to the patient identification numbers as recorded in the dataset. Data for B3GNT 6, 7, 8, 9 and ST3GAL 3 were not available in this dataset. Note that B3GalT3 mainly shows a GalNAc transferase activity in the Gb4Cer globoside synthesis (P antigen).

GCnT1	Normal	Tumor	GCnT2	Normal	Tumor	GCnT3	Normal	Tumor	GCnT4	Normal	Tumor	B3GNT1	Normal	Tumor
1	166.6	225.2	1	58	5.9	1	81.9	380	1	55.2	77.9	1	1044.9	663.7
2	85.2	325.2	2	40.5	31.9	2	40.8	309.1	2	40.1	18.5	2	1291.4	678.1
3	147.1	295	3	6.8	39	3	46.9	203.1	3	53.9	41.6	3	949.6	916.5
4	120	144.4	4	5.3	5.2	4	23.8	715	4	87.7	25.1	4	1185.7	972.7
5	155.6	372.6	5	76	64.8	5	12.1	369.9	5	9.3	46.3	5	712	823.5
6	236.3	110.6	6	30.5	49.8	6	130.7	305.3	6	169.8	68.1	6	1459.3	517.5
7	39.4	211.7	7	49.8	4.6	7	71.6	615.4	7	74.3	57.8	7	844	524.8
8	163.4	278.4	8	54.7	48.9	8	16.7	528.3	8	50	65.9	8	747.4	465.5
12	73.2	266.3	12	68.4	88.3	12	40.4	537.5	12	52.4	90.9	12	633.8	533.2
13	20.4	34.3	13	25.5	9.4	13	39.2	586.3	13	46.9	178.1	13	1022.9	1113.8
14	152.3	90.2	14	10.2	91.2	14	164.5	168.7	14	65.3	115.8	14	565.5	581.7
15	66.4	22.7	15	79.7	135.9	15	42.6	139.5	15	57	131.9	15	1125.6	813.9
16	64.4	222.2	16	3.3	4.9	16	46.5	152.9	16	33.1	28.8	16	578.2	514
18	215.6	83.6	18	87.6	94	18	67.3	324.1	18	125.2	46.2	18	791.4	813.1
19	195.3	152.4	19	39.2	65.9	19	146.2	193.9	19	249.4	91.5	19	400.5	333.3
20	156	171.5	20	78.5	188.4	20	30.8	16.8	20	79.7	19.7	20	489	872.9
21	311.5	31.2	21	32.1	130.4	21	60.8	198.9	21	173.5	116.9	21	912.9	796.7
22	197.5	59.8	22	53.6	12.8	22	61.1	112.8	22	42.6	58.5	22	894.7	631.2
23	171.3	289.3	23	55.2	204.6	23	83.2	287.6	23	122.4	45.1	23	839.4	594
24	169.5	221.7	24	45.1	12.7	24	21.3	325.6	24	22.5	113.5	24	909.2	609.1
25	121.9	178.6	25	21	19.3	25	15.5	163.8	25	70.2	87.3	25	712.1	692.3
26	215.2	67.9	26	4.2	113.7	26	35.4	272.3	26	130.2	317.1	26	721.9	691.2
27	193.5	231.6	27	42.2	210.4	27	48.8	238.5	27	101.9	470.6	27	876.8	768.4
29	36.2	329.7	29	13.7	125	29	168.7	1997.8	29	239.4	169	29	901.6	458.8
30	124.4	325.3	30	48.8	31.8	30	47.6	2700.7	30	11	97.5	30	669.7	489.1
54	177.8	475.8	54	64.4	17.3	54	95.3	69.7	54	21.4	14.7	54	995.7	295.3
57	136.1	237.1	57	51.9	18.1	57	107	1017.9	57	58.6	73.4	57	584.7	382.3

Table S2 (continuing)

B3GNT2	Normal	Tumor	B3GNT3	Normal	Tumor	B3GNT4	Normal	Tumor	MGAT3	Normal	Tumeur	POMGNT1	Normal	Tumor
1	267.8	235	1	66.8	100.2	1	111.3	147.6	1	89.7	53	1	240.1	289.5
2	225.4	171.3	2	31.6	159.6	2	163	311.5	2	89.1	65.9	2	262.6	388.9
3	578.2	438.9	3	42.6	325.3	3	128.9	125.7	3	57.1	97.7	3	262.3	254.4
4	293	317	4	28.4	207	4	105	126.9	4	143.5	162.7	4	239.7	208.5
5	24.2	29.9	5	25	249.1	5	142.4	179.8	5	22	32.4	5	275.3	393.2
6	12.6	172.2	6	47	378.8	6	212.5	129.9	6	101.1	20	6	388.1	213.4
7	335.2	30.4	7	26.7	150	7	94.1	184.2	7	53.9	21.8	7	340.2	271.9
8	457.5	264	8	66.2	215.1	8	103.7	143.4	8	117.3	57.8	8	265.7	260.4
12	264.4	58.3	12	50.3	856.2	12	137.1	210.5	12	64.8	94.6	12	284.8	191
13	243.9	37.3	13	65.2	121.2	13	244	543.2	13	61.5	180.8	13	335.1	242.2
14	172.7	33.1	14	165.9	175.7	14	221	364.2	14	144.3	42.4	14	173.8	414.5
15	372.4	381.3	15	117.6	62.1	15	220.3	256.7	15	346.3	78.7	15	297.4	183.7
16	685.7	260.4	16	21.5	81.3	16	109.5	103.4	16	103.5	69.1	16	239.9	295.3
18	436.3	489.7	18	33.1	305.3	18	211.2	214.1	18	31.7	27.9	18	412	400.8
19	278.3	211.9	19	159.7	80.4	19	180.9	222.7	19	147.8	74.4	19	508.7	490
20	280.5	248.7	20	149.4	142.8	20	144.2	318	20	36.6	58.1	20	338.8	344.3
21	57.8	27.6	21	76.6	542.8	21	292.9	413.6	21	35.6	86.6	21	531.7	544.3
22	287.2	316.6	22	53.1	145	22	212	144.2	22	22.9	103.5	22	382.2	378.8
23	414.2	430.3	23	20.5	492.6	23	160.7	459.1	23	73.5	72.8	23	335	525.5
24	467.7	159.7	24	84.5	496.6	24	142.1	201.5	24	18.4	28.5	24	398.2	251.7
25	458.7	257.1	25	19.7	82.1	25	198.4	147.5	25	93.8	27.3	25	303.2	342.6
26	342.1	192.3	26	126	240.6	26	210.9	391.6	26	36.8	36.2	26	342.4	396.1
27	704.9	82.7	27	87.8	569.6	27	238.7	925.6	27	64.2	68.9	27	254.6	441.6
29	292.5	830.1	29	55.1	225.4	29	164	420.6	29	48.6	396.2	29	224.2	237.9
30	429	279.3	30	15.9	203.2	30	85.5	123.8	30	58.5	70.7	30	282.9	271.1
54	431	302.2	54	24.8	726	54	91	93.1	54	137.1	113.2	54	273	352.4
57	379.4	156	57	27.1	163.3	57	104.6	249.7	57	89.9	92.5	57	289	452.8

Table S2 (continuing)

A4GNT	Normal	Tumor	B3GalT1	Normal	Tumor	B3GalT2	Normal	Tumor	B3GalT3	Normal	Tumor	B3GalT4	Normal	Tumor
1	120.2	113.6	1	11.5	5	1	10.4	4.7	1	207.8	48.1	1	403.1	198.6
2	33.3	68.3	2	8.3	4.3	2	40.3	1.8	2	133.9	86.7	2	560.1	345
3	160.1	164.4	3	46.8	7.6	3	33.4	16	3	138.4	79.5	3	352.9	307.8
4	170.5	232.3	4	8.4	20.2	4	69.9	1.7	4	167.8	30.1	4	496.1	340.5
5	69.1	177.6	5	58.5	10.7	5	10.7	0.8	5	76.5	100	5	409.3	374.5
6	219.8	167.8	6	36.9	25.2	6	22.8	0.7	6	120.1	58.2	6	644.9	422
7	73.8	149.6	7	3.6	6	7	21.6	22.5	7	216.8	59.8	7	310.1	435
8	127.1	107	8	7.4	15.3	8	25.2	11.9	8	153.6	52	8	340.8	367.8
12	325.4	183.8	12	14.8	6.5	12	9.1	1.3	12	127.9	118.4	12	363.3	404.8
13	132.9	40.6	13	22.5	102.4	13	5.9	3.1	13	53.2	61.6	13	498.5	1050.9
14	202.9	324	14	18.1	11	14	6.3	6.7	14	165.7	4	14	382.4	655.6
15	322.7	290.4	15	15.5	20	15	4.4	6.8	15	112.1	93.2	15	364.7	390.3
16	81.1	107.8	16	11.9	5.9	16	3.6	4.3	16	125.1	104.4	16	147.1	263.8
18	100.3	216.7	18	10.1	6.5	18	26.6	2.3	18	244.4	113.9	18	358.4	387.9
19	562.8	152.4	19	81	3.9	19	49.8	1.5	19	180.3	4.7	19	536.5	397.5
20	287.2	318.6	20	5.8	23.1	20	3.1	81.2	20	98	256.3	20	260.2	413.9
21	261.7	302.5	21	7.5	65.7	21	11.8	5	21	84.9	72.7	21	342.1	374.2
22	98.9	168.4	22	39.8	8.4	22	37.3	2.9	22	153	92.4	22	457.5	177.1
23	181.5	410.5	23	7.2	48.4	23	29.7	16.1	23	139.3	41.3	23	272.5	318.3
24	136.4	151.8	24	5	10.2	24	42.4	4.2	24	306.5	21.8	24	346.4	126.6
25	131.6	147.6	25	6.2	5.1	25	19.5	4	25	238.3	9	25	265.5	287.8
26	175.3	251.7	26	5.8	12.4	26	31.7	3.8	26	98.5	30.1	26	372.7	440.5
27	174.4	566.2	27	7.6	57.9	27	50.3	9.6	27	164	15.3	27	834	488.7
29	220.8	176.5	29	15.8	10.7	29	38.2	2.2	29	125.5	92.5	29	375.9	408
30	120.3	126.8	30	4.5	2.8	30	64.8	1.9	30	160.3	66.4	30	400.1	393.7
54	125.5	63.9	54	7.2	4.7	54	19	1.2	54	163.6	98.3	54	454.3	216.3
57	123.2	110.2	57	36.5	7.7	57	42.9	0.7	57	64.2	17.1	57	319.6	146.6

Table S2 (continuing)

B3GalT5	Normal	Tumor	B3GalT5	Normal	Tumor	B4GalT2	Normal	Tumor	B4GalT3	Normal	Tumor	B4GalT4	Normal	Tumor
1	66.7	46	1	66.7	46	1	366.6	414.4	1	509.7	536.6	1	63.6	250.5
2	94	45	2	94	45	2	366.3	501.1	2	474.1	560.5	2	151.4	242.4
3	111.3	37.8	3	111.3	37.8	3	282.2	530.1	3	692.6	762.2	3	142.3	207.4
4	120.6	83.4	4	120.6	83.4	4	468.1	455.6	4	513.5	915	4	125.9	95.5
5	120.9	87.8	5	120.9	87.8	5	424.4	627.8	5	795.3	830	5	150	129.3
6	139.4	69.6	6	139.4	69.6	6	491	446.6	6	569.9	749.4	6	23.5	149.4
7	98.5	113.8	7	98.5	113.8	7	349.6	499.1	7	617	620.1	7	135.6	177.6
8	136.3	71.2	8	136.3	71.2	8	431.9	522.4	8	691.7	844.8	8	176.3	129.7
12	98.5	193.9	12	98.5	193.9	12	618.8	946.4	12	708.5	1162.8	12	112.6	169.5
13	52.5	32.5	13	52.5	32.5	13	941.6	1155.7	13	551.2	1195.5	13	55.6	31.2
14	94.6	135.9	14	94.6	135.9	14	788.7	1197	14	674.8	1485.8	14	128.1	86
15	28.7	181.2	15	28.7	181.2	15	736.7	645	15	421.1	713.9	15	91.5	200.4
16	37.5	47.2	16	37.5	47.2	16	415.4	483.8	16	634.6	528.7	16	170.1	196.1
18	111.2	127.8	18	111.2	127.8	18	391.4	466.9	18	454.9	1059.4	18	163.7	204.3
19	162.5	121.2	19	162.5	121.2	19	575	506.7	19	785.7	519	19	83.7	130
20	103.5	362	20	103.5	362	20	437.5	631.1	20	554.2	740.3	20	122.1	196.8
21	156.6	203.4	21	156.6	203.4	21	782.1	754.6	21	793.6	1153.4	21	166.9	208.5
22	123.5	68.9	22	123.5	68.9	22	386.9	316.2	22	504.3	563.3	22	139.4	240.1
23	16.8	50.3	23	16.8	50.3	23	364.7	608.4	23	489.3	744.8	23	225	5.9
24	48.7	128.2	24	48.7	128.2	24	485.5	821.9	24	608.7	523.5	24	78.1	224.1
25	99.2	19.5	25	99.2	19.5	25	332.6	344.5	25	593.3	677	25	167.7	197.2
26	140	164.9	26	140	164.9	26	240.3	723.7	26	523.2	744.3	26	114.6	101.4
27	164.5	501.8	27	164.5	501.8	27	490.3	970.2	27	497.7	1043.2	27	142.5	35
29	96.7	86.5	29	96.7	86.5	29	292.4	561.8	29	507.5	1100.1	29	104.8	188
30	46.4	99.5	30	46.4	99.5	30	347.8	480.3	30	535.1	728.1	30	110.8	180.5
54	91.8	26	54	91.8	26	54	333.7	883.6	54	485.1	1042	54	87.6	105.3
57	14	116.9	57	14	116.9	57	308.7	500.1	57	450.5	919	57	99.8	80.4

Table S2 (continuing)

B4GalT5	Normal	Tumor	B4GalT6	Normal	Tumor	B4GalT7	Normal	Tumor	ST3Gal1	Normal	Tumor	ST3Gal2	Normal	Tumor
1	805.4	1318.4	1	12.7	11.5	1	312.2	252.3	1	23.1	66.5	1	8.1	49.4
2	2243.6	1901.8	2	34	11.1	2	204.3	420.5	2	129.1	16.2	2	4.8	12.8
3	2026.2	2764.7	3	11.2	63.7	3	112.4	357.2	3	75.4	43.1	3	78.9	95.4
4	802	1538.1	4	6.3	11.3	4	210.3	233.4	4	13.3	43.5	4	86.6	53.8
5	2327.2	1405.1	5	7.1	7.1	5	270.3	349.4	5	93.1	16.8	5	78.7	62.2
6	921.5	1303.1	6	7.8	13.7	6	262.9	286.4	6	74.6	125.3	6	16.2	69
7	1688.9	2025.8	7	7	42.2	7	172.4	420	7	18.7	85.9	7	18.8	53.5
8	1380.3	1981.8	8	12.6	3.4	8	135.3	427.6	8	84	112.3	8	30.5	45
12	1936.1	1873.9	12	12.9	13.3	12	303	162	12	10.3	27.5	12	39.6	27.7
13	953.2	1488.3	13	95.1	100.4	13	403.7	853.5	13	43.2	546.9	13	116.8	267.2
14	933.2	823.2	14	17.1	155.5	14	292.7	528.6	14	10.2	124.3	14	30.6	196.9
15	2112.8	1487.4	15	10.6	15.3	15	197.4	237.2	15	14.9	228.5	15	45.2	117.8
16	2222.2	1205.4	16	17.3	4.9	16	150.9	223.8	16	125.9	48.2	16	9.6	51.7
18	1164.4	1581.3	18	11.7	23.5	18	272.7	522.6	18	63.4	5.6	18	11.5	38
19	1984.2	1333.1	19	11.3	15.2	19	302.1	396.6	19	251.3	81.9	19	209.2	95.7
20	1112.7	902.1	20	32.4	16.9	20	284.1	339.8	20	84.9	172.7	20	62.7	129.2
21	823.8	1147.1	21	6.3	39.2	21	334.4	695.1	21	180.1	36.1	21	70.1	57.6
22	991.5	1253.3	22	12.9	4.8	22	278.1	271.8	22	66.6	53.2	22	20.3	91
23	1126.3	1275.7	23	48.1	16.6	23	289	827.8	23	69.2	131.8	23	77.6	29.6
24	922	916.5	24	30.5	4.5	24	182.9	307	24	84.8	91.4	24	14.1	16.8
25	1463.9	1566.4	25	4.6	16.6	25	223	308	25	107.8	213.9	25	35.5	40.5
26	1160.5	1819.9	26	59	12.2	26	171.8	418.7	26	17.4	11.3	26	58.1	15
27	989.5	935.9	27	65	32.1	27	218.6	59.2	27	19.9	23.7	27	44.4	54
29	1296.1	1114.7	29	6.8	74.1	29	236.9	378.8	29	62.7	119.5	29	35.6	50.1
30	1255.8	955.5	30	18	33.5	30	176.6	312.3	30	46.8	146.6	30	36.2	47.9
54	1671	2556.6	54	14.7	12.1	54	322.3	343.2	54	63.4	257.3	54	79.1	39.9
57	1938.2	2110.5	57	11.3	8.5	57	194	452.8	57	109.7	9.6	57	79.8	109

Table S2 (continuing)

ST3Gal4	Normal	Tumor	ST3Gal5	Normal	Tumor	ST3Gal6	Normal	Tumor
1	63.6	250.5	1	1539.4	1509.8	1	167.5	296.5
2	151.4	242.4	2	1956	1880.2	2	187.6	165.6
3	142.3	207.4	3	1162.3	2303.5	3	136.1	98
4	125.9	95.5	4	1459.5	1567.6	4	127.2	201.4
5	150	129.3	5	1141.4	3560	5	49.5	87.2
6	23.5	149.4	6	1690.9	2340	6	223	115.6
7	135.6	177.6	7	1600.9	3911.7	7	116.2	145.4
8	176.3	129.7	8	1365.8	1986.8	8	171.8	93.5
12	112.6	169.5	12	853.7	721.1	12	254.2	119.4
13	55.6	31.2	13	2137.4	1362.5	13	268.3	78.6
14	128.1	86	14	1314.2	2341.3	14	93.2	19.2
15	91.5	200.4	15	991.1	1391.1	15	189.1	208
16	170.1	196.1	16	1413.6	1241.5	16	192.9	163.6
18	163.7	204.3	18	1563.6	1147.7	18	215.8	94.4
19	83.7	130	19	1235.9	552.8	19	64.6	81.2
20	122.1	196.8	20	1071.4	1045.9	20	148.7	366.4
21	166.9	208.5	21	1930	2449.2	21	184.4	226.9
22	139.4	240.1	22	1537.5	2289.1	22	168	403.6
23	225	5.9	23	1607.3	1028.6	23	256	576.2
24	78.1	224.1	24	1611.4	3020.7	24	132.3	71.9
25	167.7	197.2	25	976.7	1666.5	25	157.1	134.2
26	114.6	101.4	26	1969	2316.9	26	191.8	261.5
27	142.5	35	27	1910.9	656.9	27	260.6	24.1
29	104.8	188	29	1192.3	561.3	29	104.5	8.4
30	110.8	180.5	30	1182.1	1303.4	30	132.9	276.8
54	87.6	105.3	54	1142.2	698.5	54	179.6	42.9
57	99.8	80.4	57	1354.1	1409	57	90.4	77.5
