# Supplementary material for: A Recombinant Fungal Lectin for Labeling Truncated Glycans on Human Cancer Cells
Source: PLoS One. 2015 Jun 4;10(6):e0128190. doi: 10.1371/journal.pone.0128190 (PMC4456360; doi:10.1371/journal.pone.0128190)
Supplement: S3 Table — Binding of rPVL was determined by flow cytometry on cancer cell lines from various histological origins as described in Material and Methods. ND: not determined. (PDF) [file pone.0128190.s008.pdf]

Table S3: rPVL binding on various cancer cells

Binding of rPVL was determined by flow cytometry on cancer cell lines from various histological origins as described in Material and Methods. ND: not determined

Cell line	Origin	PVL staining	Inhibtion by GlcNac	Inhibition by Sialidase
H322	Lung adenocarcinoma	+++	ND	ND
H441	Lung adenocarcinoma	+++	ND	ND
A431	Skin squamous cell carcinoma	++	ND	ND
A375	Melanoma	++	ND	-
Colo829	Melanoma	+++	ND	ND
SKMel28	Melanoma	++	ND	ND
M119	Melanoma	++	++	ND
HT-29	Colorectal adenocarcinoma	+++	+++	ND
DU-145	Prostate carcinoma	+++	+++	ND
MDA-MB-231	Breast carcinoma	+	ND	ND
OVCAR3	Ovary adenocarcinoma	++	++	ND
CHO	Ovary (hamster)	+++	+++	+
