# Supplementary material for: A Recombinant Fungal Lectin for Labeling Truncated Glycans on Human Cancer Cells
Source: PLoS One. 2015 Jun 4;10(6):e0128190. doi: 10.1371/journal.pone.0128190 (PMC4456360; doi:10.1371/journal.pone.0128190)
Supplement: S4 Table — A TMA prepared with tissues from various origins coming from 10 different donors was stained with rPVL-biot at 0,7 μg/ml as such or in presence of 0,1 M of fucose or N-acetyglucosamine. Digestive tissues showed the strongest staining; some of it could not be completely removed even in presence of an excess of free GlcNAc. (PDF) [file pone.0128190.s009.pdf]

Table S4: PVL staining of healthy tissues. A TMA prepared with tissues from various origins coming from 10 different donors was stained with rPVL-biot at 0,7 µg/ml as such or in presence of 0,1 M of fucose or N-acetyglucosamine. Digestive tissues showed the strongest staining; some of it could not be completely removed even in presence of an excess of free GlcNAc.

**Donor**	**Tissue type**	**PVL Staining**		
		Alone	+Fuc	+GlcNac
5975	Esophagus + cardia	2	2	1
6064	Esophagus + cardia	2	2	1
6064	Pylorus + duodenum	3	3	2
6064	Vagina	1 (Keratinocytes)	1	0
6064	Ovary	0	0	0
5971	Jejunum	2	2	1
5971	Right side Colon	2	2	1
5971	Transverse colon	1	1	0
6022	Trachea + Esophagus+ lymph node	1 (Tracheal glands)	1	0
6183	Cervix	1	0	0
6194	Pancreas	1	1	0
6444	Liver	1 (Diffuse)	1	0
6444	Ovary	0	0	0
6444	Oviduct, Fallopian tubes	1 (Apical side of the epithelium)	1	0
6444	Endometer	1	1	0
6450	Trachea + lymph node	1	1	0
6074	Endometer	0	0	0
6022	Pylorus + duodenum	3	3	2
